# ZDHHC9‐Mediated Palmitoylation of ACSL4 Drives Ferroptosis in Diabetes Mellitus–Induced Erectile Dysfunction

**DOI:** 10.1002/advs.202517067

**Published:** 2026-05-05

**Authors:** Wanyang Guo, Ming Xiao, Mengjun Huang, Dongzi Peng, Ruijiang Zeng, Ruilin Liu, Yuanqiao Zhao, Zhihan Ouyang, Yulong Hong, Zexian Ding, Zhuo Xing, Hao Su, Jinxiang Wang, Wenjun Mao, Xin Jin

**Affiliations:** ^1^ Department of Urology The Second Xiangya Hospital Central South University Changsha Hunan China; ^2^ Key Laboratory of Diabetes Immunology (Central South University) Ministry of Education National Clinical Research Center for Metabolic Disease Changsha China; ^3^ FuRong Laboratory Changsha Hunan China; ^4^ Biobank of the Second Xiangya Hospital of Central South University Changsha Hunan China; ^5^ Department of Gastroenterology The Second Xiangya Hospital Central South University Changsha Hunan China; ^6^ Department of Urology Kidney and Urology Center Pelvic Floor Disorders Center The Seventh Affiliated Hospital Sun Yat‐Sen University Shenzhen Guangdong China; ^7^ Department of Thoracic Surgery The Affiliated Wuxi People's Hospital of Nanjing Medical University, Wuxi People's Hospital Wuxi Medical Center Nanjing Medical University Wuxi Jiangsu China

**Keywords:** ACSL4, DMED, metabolic disorder, palmitoylation, ZDHHC9

## Abstract

Diabetes mellitus‐induced erectile dysfunction (DMED) is a highly prevalent complication among diabetic patients; however, its underlying pathogenic mechanisms remain incompletely understood. Metabolic disorder is a hallmark of diabetes, yet its precise contribution to DMED progression is not well defined. In this study, we demonstrate that metabolic disturbances, particularly elevated levels of palmitic acid (PA), induce ferroptosis in corpus cavernosum fibroblasts, thereby contributing to the development of erectile dysfunction. Mechanistically, we identified ZDHHC9, a palmitoyltransferase, to be aberrantly upregulated in DMED, where it catalyzes the S‐palmitoylation of ACSL4 at cysteine 595. This post‐translational modification enhances ACSL4 enzymatic activity, promotes lipid peroxidation, and drives ferroptosis in fibroblasts. Furthermore, we found that hyperactivation of the PI3K/AKT signaling pathway serves as a key upstream regulator of ZDHHC9 expression in this context. To explore the therapeutic potential of targeting this pathway, we developed siRNA against Zdhhc9 encapsulated in lipid nanoparticles (siZdhhc9‐LNPs), which effectively suppressed Zdhhc9 expression in the corpus cavernosum and ameliorated erectile dysfunction in DMED mice. Collectively, our findings reveal a pathological cascade linking metabolic dysregulation to fibroblast ferroptosis via ZDHHC9‐mediated ACSL4 palmitoylation, and establish ZDHHC9 as a promising therapeutic target for the treatment of DMED.

## Abstract 2

This study reveals that metabolic dysregulation in diabetes activates the ZDHHC9‐ACSL4 pathway, triggering ferroptosis in penile fibroblasts and worsening erectile dysfunction. Targeting ZDHHC9 with siRNA‐loaded lipid nanoparticles alleviates this condition in mice, offering a novel therapeutic strategy for diabetes‐induced sexual dysfunction through ferroptosis modulation.

## 1 Introduction

Diabetes mellitus is a well‐documented independent risk for erectile dysfunction (ED), with epidemiological studies consistently demonstrating that diabetic patients experience earlier onset, more severe symptoms, and diminished therapeutic responsiveness compared to non‐diabetic individuals [1, 2, 3]. While current pathophysiological research predominantly emphasizes endothelial dysfunction and smooth muscle cell (SMC) impairment in DMED progression, the role of penile fibroblasts—constituting over 60% of corpus cavernosum stromal cells—has remained critically underexplored despite their emerging regulatory roles in erectile physiology [4].

Metabolic dysregulation serves as a central pathogenic nexus in DMED development [5]. Glucose and lipid dyshomeostasis stand as particularly salient contributors. Lipid metabolic abnormalities exacerbate DMED pathogenesis by inducing regulated cell death processes, notably ferroptosis—a genetically encoded, iron‐dependent form of programmed cell death distinguished by iron overload, lipid peroxidation, and compromised antioxidant defenses [6, 7, 8, 9]. Dysregulation of the balance between lipid peroxide generation and clearance has been implicated in DMED models, with studies reporting iron overload, oxidative stress, and downregulation of ferroptosis inhibitors (e.g., GPX4, SLC7A11) in diabetic animal corpora cavernosa [10, 11, 12]. Recent evidence further demonstrates that fibroblasts—key mediators of extracellular matrix (ECM) remodeling—undergo ferroptosis in diabetic contexts, contributing to impaired wound healing and tissue fibrosis [13, 14]. However, the role of fibroblast ferroptosis in DMED pathophysiology remains uncharted.

Lipid metabolic dysregulation profoundly impacts post‐translational protein modifications (PTMs), particularly S‐palmitoylation—a reversible lipidation process where palmitic acid (PA) is covalently attached to cysteine residues via thioester bonds [15]. This dynamic modification, orchestrated by ZDHHC family acyltransferases and reversed by acyl protein thioesterases (APTs), critically regulates protein stability, subcellular localization, and functional activity [16]. Emerging evidence highlights its dysregulation in metabolic pathologies, where lipid‐derived intermediates accumulate to drive aberrant lipidation [17, 18, 19]. Palmitoylation also emerges as a pivotal modulator of ferroptosis, an iron‐dependent programmed cell death driven by lipid peroxidation. For instance, ZDHHC20‐mediated palmitoylation at cysteine 66 (Cys66) stabilizes glutathione peroxidase 4 (GPX4) by enhancing its membrane anchoring and catalytic efficiency, thereby suppressing ferroptosis and promoting tumor cell survival [20]. Similarly, ZDHHC8 compensates by modifying GPX4 at Cys75, protecting against CD8^+^ T cell‐induced ferroptosis through enhanced phospholipid hydroperoxide detoxification [21]. However, the reciprocal regulation between palmitoylation and ferroptosis remains incompletely understood. ACSL4 has not yet been shown to undergo palmitoylation‐mediated regulation. Given ACSL4's central role in lipid peroxidation capacity [22], investigating its PTM‐based modulation represents a critical gap in understanding metabolic control of ferroptosis in diabetes‐associated pathologies.

In this study, we identify ZDHHC9—a DHHC family palmitoyltransferase—as the enzyme responsible for S‐palmitoylation of ACSL4 at cysteine residue 595 (Cys595). This post‐translational modification enhances ACSL4's catalytic activity, amplifying lipid peroxidation capacity and sensitizing fibroblasts to ferroptosis. Mechanistically, aberrant PI3K/AKT hyperactivation acts as the predominant upstream driver of ZDHHC9 upregulation in penile fibroblasts, a previously unrecognized pathological consequence of metabolic dysregulation in DMED. Furthermore, pharmacological inhibition of ZDHHC9 or site‐specific mutation of ACSL4 Cys595 abrogates ferroptosis in diabetic penile fibroblasts, underscoring the functional significance of this regulatory axis. By revealing a previously uncharacterized role for ZDHHC9‐mediated ACSL4 palmitoylation in metabolic‐oxidative stress crosstalk, our work establishes this pathway as a druggable target for preserving fibroblast integrity and restoring erectile function in diabetic patients.

## 2 Results

### 2.1 Elevated Palmitic Acid Levels as a Characteristic Metabolic Alteration in Patients with DMED

**FIGURE 1:**
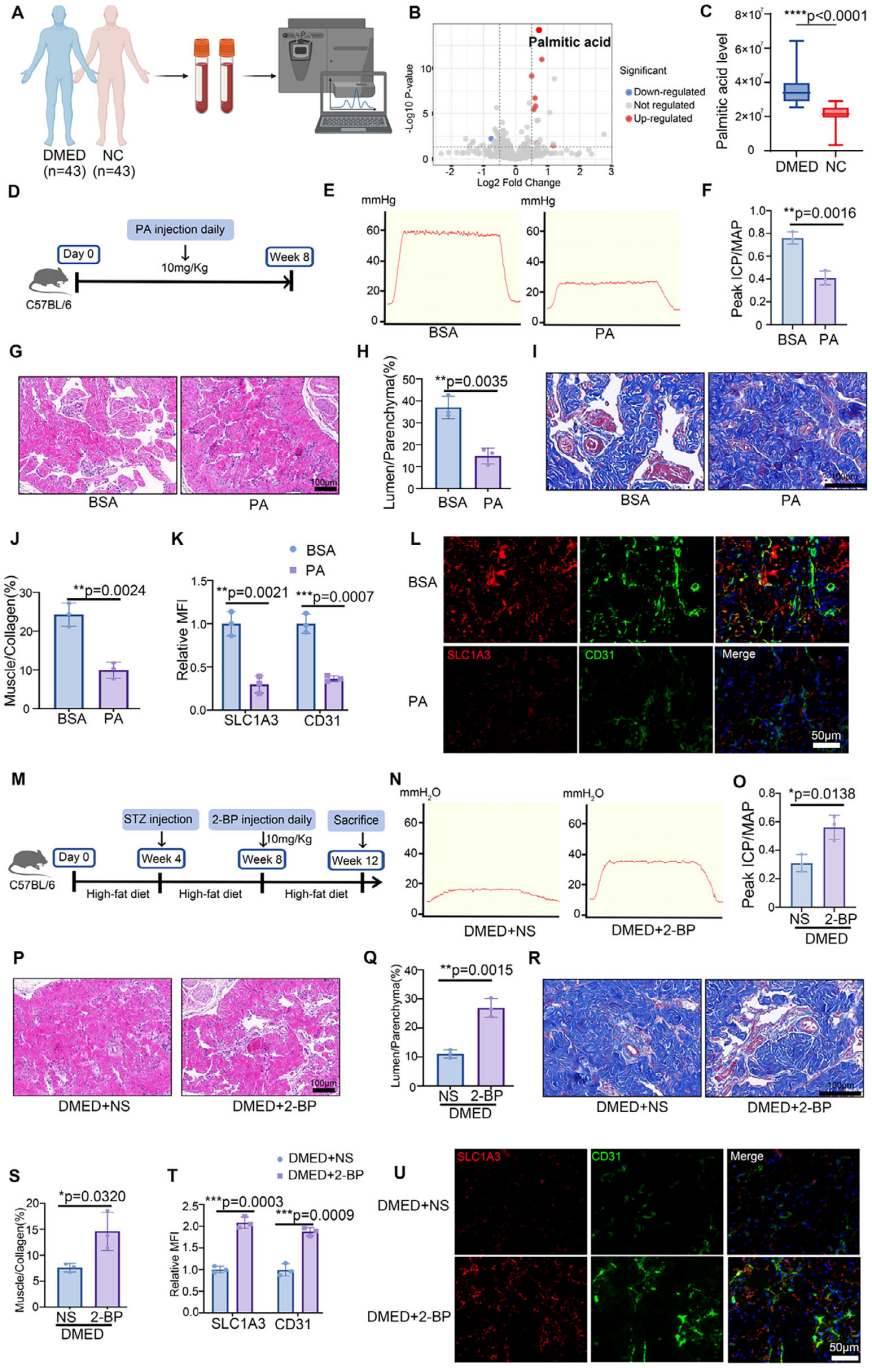
Metabolic disorder–induced elevation of palmitic acid (PA) contributes to DMED pathogenesis. (A) Schematic diagram. (B) Volcano plot illustrating differential metabolite expression in the untargeted metabolomics analysis. (C) Box plot showing elevated PA levels in the DMED group compared with the NC group (*n* = 43). (D) Experimental design of PA injection in vivo. (E,F) Representative images of ICP and quantification of ICP/MAP ratio. (*n* = 3). (G,H) Morphology and quantification of the corpus cavernosum assessed by H&E staining. Scale bars, 100 µm (*n* = 3). (I,J) Collagen (blue) and smooth muscle (red) evaluated by Masson staining. Scale bars, 100 µm (*n* = 3). (K,L) Representative images and quantification of Slc1a3 (red) and CD31 (green) immunofluorescence staining. Scale bars, 50 µm (*n* = 3). (M) Experimental design of DMED model construction and 2‐BP injection in vivo. (N,O) Representative images of ICP and quantification of ICP/MAP ratio (*n* = 3). (P,Q) Morphology and quantification of the corpus cavernosum assessed by H&E staining. Scale bars, 100 µm (*n* = 3). (R,S) Collagen (blue) and smooth muscle (red) evaluated by Masson staining. Scale bars, 100 µm (*n* = 3). (T,U) Representative images and quantification of Slc1a3 (red) and CD31 (green) immunofluorescence staining. Scale bars, 50 µm (*n* = 3). Box plots (C) show the median and IQR, and whiskers represent the full data range (minimum to maximum). Bar charts (F,H,J,K,N,Q,S,T) are presented as mean ± SD. All experiments were performed with at least three biologically independent cell/mouse samples with similar results. Unpaired two‐sided Student's *t*‐test (C,F,H,J,K,N,Q,S,T) was performed. *p*‐values have been indicated in the figures, and *p* < 0.05 is considered statistically significant.

To elucidate the metabolic disorder underlying DMED, we conducted untargeted metabolomic profiling of clinical plasma samples (Figure 1A). Pronounced metabolic alterations were observed in patients with DMED (Figure 1B), with PA levels significantly elevated compared to healthy controls (Figure 1C). Consistently, PA levels were also markedly increased in penile cavernous tissue homogenates of DMED mice (Figure S1A). Then, a mouse model of chronic PA exposure was established [23] (Figure 1D). PA‐treated mice exhibited a marked reduction in intracavernosal pressure (ICP)/mean arterial pressure(MAP) ratio, indicating impaired erectile function (Figure 1E,F). Histological analysis revealed decreased cavernosal sinusoidal space‐to‐parenchyma and smooth muscle‐to‐collagen ratios, reflecting smooth muscle atrophy and fibrosis (Figure 1G–J). To assess PA's impact on functional cell populations in the corpus cavernosum, immunofluorescence staining for endothelial cells (CD31) and fibroblasts (SLC1A3) was performed. Both markers showed reduced fluorescence intensity in the corpus cavernosum of PA‐exposed mice (Figure 1K,L), indicating endothelial dysfunction and fibroblast depletion. These findings demonstrate that chronic PA exposure directly compromises cavernosal architecture and erectile function in vivo.

Given that S‐palmitoylation is a critical posttranslational modification mediating the pathological effects of PA [24], we sought to determine whether aberrant palmitoylation contributes to PA‐induced DMED. DMED mice were treated with 2‐bromopalmitate (2‐BP), a broad‐spectrum palmitoylation inhibitor (Figure 1M). 2‐BP administration partially restored ICP (Figure 1N,O), improved sinusoidal space and smooth muscle–to–collagen ratios (Figure 1P–S), and enhanced endothelial and fibroblast populations in cavernosal tissue (Figure 1T,U). These results highlight aberrant palmitoylation as a pathogenic mechanism in DMED and suggest that targeting this posttranslational modification could mitigate functional and structural impairments.

### 2.2 Upregulation of Zdhhc9 Drives DMED Progression in Mice

**FIGURE 2:**
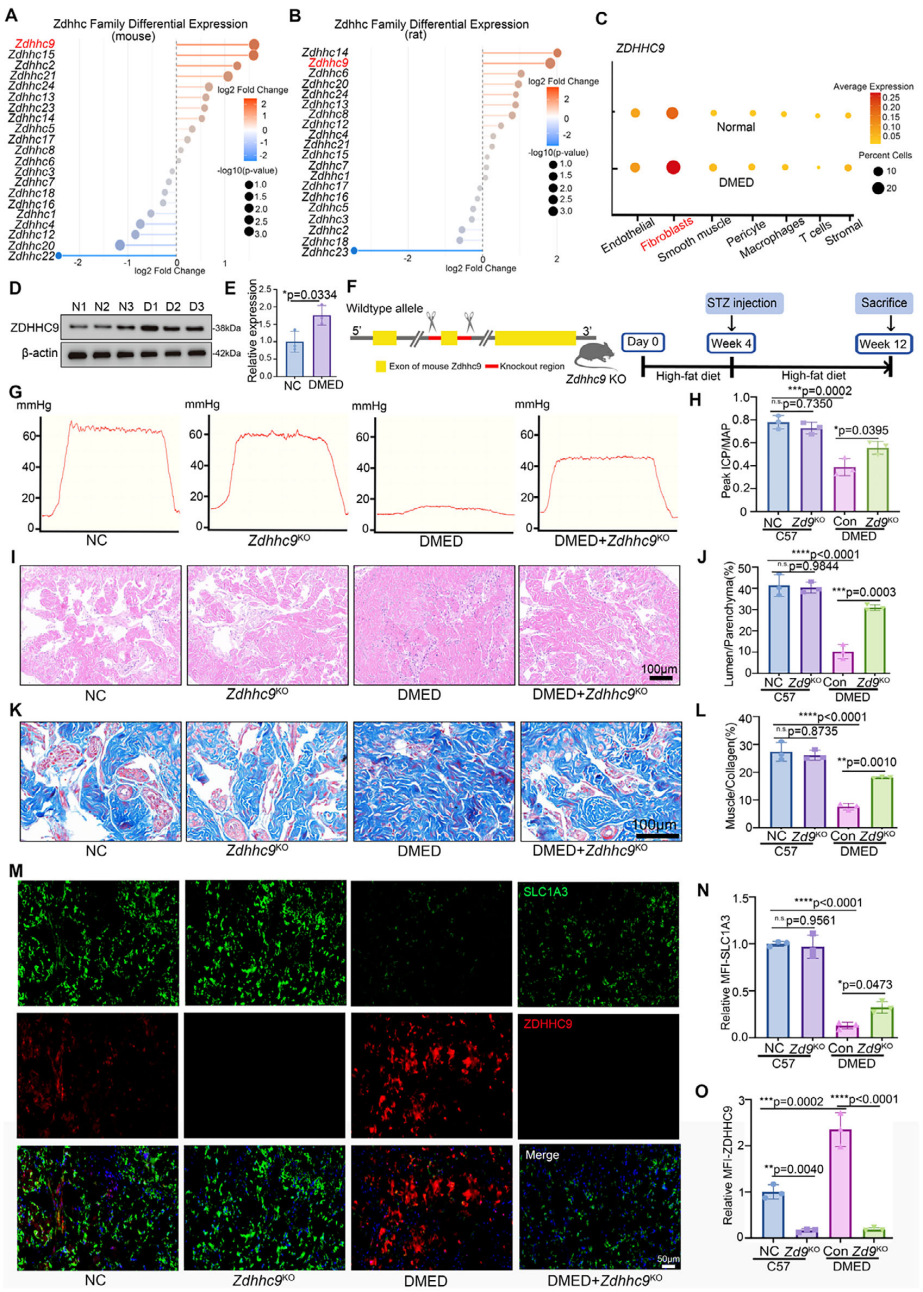
Upregulation of ZDHHC9 drives DMED progression in mice. (A) Lollipop chart showing transcriptome sequencing of the *Zdhhc* family in mice. (B) Lollipop chart showing transcriptome sequencing of the *Zdhhc* family in rats. (C) Expression of ZDHHC9 in each group based on single‐cell data analysis of the human corpus cavernosum (http://malehealthatlas.cn/). (D,E) Protein expression of ZDHHC9 in normal and DMED mice (*n* = 3). (F) Experimental design of Zdhhc9 knockout (Zdhhc9^ko^) mice model construction and DMED model induction. (G,H) Representative images of ICP and quantification of ICP/MAP ratio (*n* = 3). (I,J) Morphology and quantification of the corpus cavernosum assessed by H&E staining. Scale bars, 100 µm (*n* = 3). (K,L) Collagen (blue) and smooth muscle (red) evaluated by Masson staining. Scale bars, 100 µm (*n* = 3). (M–O) Representative images and quantification of SLC1A3 (green) and ZDHHC9 (red) immunofluorescence staining. Scale bars, 50 µm (*n* = 3). Bar charts(E,H,J,L,N,O) are presented as mean ± SD. All experiments were performed with at least three biologically independent cell/mouse samples with similar results. Unpaired two‐sided Student's *t*‐test (E) and one‐way ANOVA with Tukey's post hoc test (H,J,L,N,O) were performed. *p*‐values have been indicated in the figures, and *p* < 0.05 is considered statistically significant.

To identify the palmitoyl acyltransferase (PAT) driving aberrant palmitoylation in DMED, we performed comprehensive transcriptomic analyses of PAT family members in whole‐tissue RNA‐sequencing datasets from both murine and rat DMED models. Among all PAT isoforms examined, ZDHHC9 emerged as the sole member consistently upregulated in both species (Figure 2A,B). Integrative analysis of publicly available single‐cell RNA‐sequencing data from human cavernosal tissue (http://malehealthatlas.cn/) further localized *ZDHHC9* overexpression specifically to fibroblast populations in DMED patients (Figure 2C). These findings were corroborated at the protein level, with Western blotting revealing significantly elevated ZDHHC9 expression in corpus cavernosum tissues of DMED mice (Figure 2D,E). To interrogate the functional significance of *Zdhhc9* in DMED pathogenesis, we generated a *Zdhhc9*‐deficient murine model (Figure 2F). Notably, Zdhhc9 knockout (KO) DMED mice exhibited marked improvements in ICP compared to wild‐type DMED controls (Figure 2G,H). Histological assessment revealed attenuated fibrotic remodeling and preserved cavernosal architecture in *Zdhhc9* ^ko^ mice, as evidenced by restored sinusoidal space‐to‐parenchyma ratios and enhanced smooth muscle‐to‐collagen ratios (Figure 2I–L). Immunofluorescence co‐staining for ZDHHC9 and the fibroblast marker SLC1A3 further demonstrated fibroblast population recovery in Zdhhc9‐deficient corpora cavernosa (Figure 2M–O). Collectively, these findings identify ZDHHC9 as a key palmitoyltransferase upregulated in DMED and demonstrate that its deletion preserves the number of fibroblasts and improves erectile function.

### 2.3 Hyperactivation of the PI3K/AKT Signaling Mediates ZDHHC9 Upregulation in DMED

**FIGURE 3:**
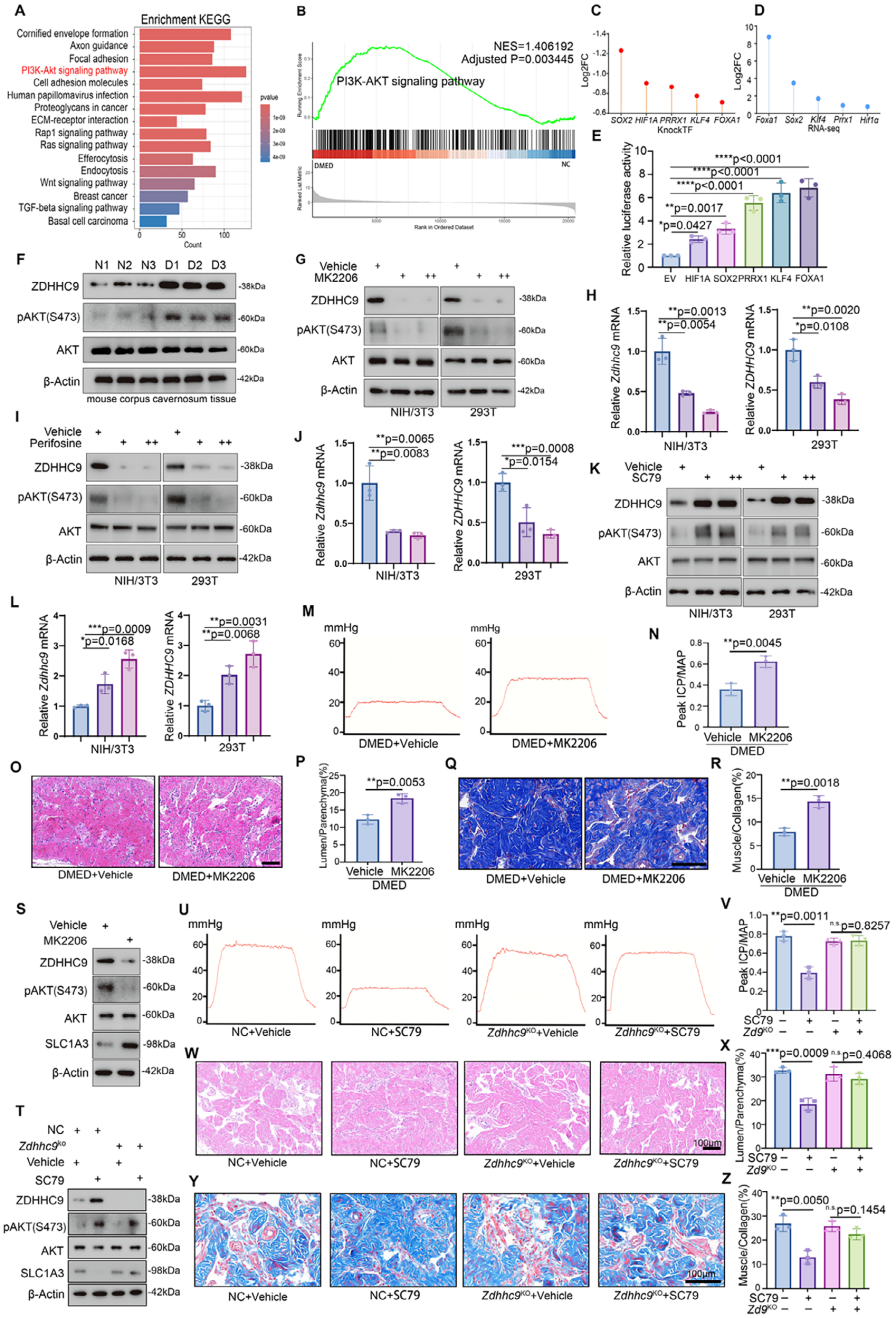
Hyperactivation of the PI3K/AKT signaling pathway mediates ZDHHC9 upregulation in DMED. (A) Bubble plot illustrating KEGG pathway enrichment analysis of transcriptomic sequencing data from corpus cavernosum tissue of DMED mice. (B) Gene Set Enrichment Analysis showing significant activation of the PI3K/AKT signaling pathway in the DMED group. (C) Lollipop plot illustrating transcription factors predicted to regulate ZDHHC9 and themselves regulated by PI3K/AKT signaling, based on the KnockTF database (http://www.licpathway.net/KnockTF/). (D) Lollipop plot showing expression levels of selected transcription factors in corpus cavernosum transcriptomic sequencing of DMED mice. (E) Luciferase reporter assay confirming the binding of indicated transcription factors to the ZDHHC9 promoter (*n* = 3). (F) Activation of the PI3K/AKT signaling pathway in corpus cavernosum tissue of DMED mice. (G,H) Effects of MK2206 treatment on ZDHHC9 levels in NIH/3T3 and 293T cells (*n* = 3). (I,J) Effects of perifosine treatment on ZDHHC9 levels in NIH/3T3 and 293T cells (*n* = 3). (K,L) Effects of SC79 treatment on ZDHHC9 levels in NIH/3T3 and 293T cells (*n* = 3). (M,N) Representative images of ICP and quantification of ICP/MAP ratio (*n* = 3). (O,P) Morphology and quantification of the corpus cavernosum assessed by H&E staining. Scale bars, 100 µm (*n* = 3). (Q,R) Collagen (blue) and smooth muscle (red) evaluated by Masson staining. Scale bars, 100 µm (*n* = 3). (S,T) Western blot analysis of MK2206 and SC79 treatments on Zdhhc9 and PI3K/AKT signaling in vivo. (U,V) Representative images of ICP and quantification of ICP/MAP ratio (*n* = 3). (W,X) Morphology and quantification of the corpus cavernosum assessed by H&E staining. Scale bars, 100 µm (*n* = 3). (Y,Z) Collagen (blue) and smooth muscle (red) evaluated by Masson staining. Scale bars, 100 µm (*n* = 3). Bar charts(E,H,J,L,N,P,R,V,X,Z) are presented as mean ± SD. All experiments were performed with at least three biologically independent cell/mouse samples with similar results. Unpaired two‐sided Student's *t*‐test (N,P,R) and one‐way ANOVA with Tukey's post hoc test (E,H,J,L,V,X,Z) were performed. *p*‐values have been indicated in the figures, and *p* < 0.05 is considered statistically significant.

To elucidate the molecular mechanisms driving ZDHHC9 overexpression in the corpus cavernosum of DMED mice, we conducted integrative pathway analyses of differentially expressed genes. KEGG pathway enrichment and GSEA both revealed significant activation of the PI3K‐AKT signaling axis (Figure 3A,B). Bioinformatics interrogation using the KnockTF database predicted multiple PI3K/AKT downstream transcription factors with potential regulatory roles in *ZDHHC9* expression. Integrated transcriptomic profiling of DMED mice identified SOX2, HIF1A, PRRX1, KLF4, and FOXA1 as candidate activators of *ZDHHC9* (Figure 3C), all showing significant upregulation in diabetic corpus cavernosum tissues (Figure 3D). ChIP‐Atlas database analysis revealed potential binding sites for these transcription factors at the *ZDHHC9* promoter region (Figure S2A–E). Functional validation through gain‐ and loss‐of‐function experiments demonstrated their regulatory effects on ZDHHC9 expression at both transcriptional and translational levels. Chromatin immunoprecipitation followed by quantitative PCR (ChIP‐qPCR) confirmed direct promoter occupancy of all five transcription factors (Figure S2F–AD). Furthermore, luciferase reporter assays substantiated their direct transcriptional regulation of ZDHHC9 (Figure 3E).

Biochemical analysis confirmed hyperphosphorylation of AKT and concomitant ZDHHC9 overexpression in DMED corpus cavernosum tissues (Figure 3F). In vitro pharmacological inhibition of PI3K/AKT signaling using MK‐2206 (AKT inhibitor) or perifosine (AKT membrane translocation inhibitor) in NIH/3T3 and HEK293T cells significantly attenuated ZDHHC9 expression at both mRNA and protein levels (Figure 3G–J). In vivo administration of MK‐2206 to DMED mice effectively suppressed PI3K/AKT signaling, reduced ZDHHC9 expression, and ameliorated erectile dysfunction as evidenced by restored ICP (Figure 3M,N; Figure S3A,B). Histological evaluation revealed decreased fibrotic remodeling and recovery of the fibroblast marker SLC1A3 in treated animals (Figure 3O–S; Figure S3C–E). Perifosine treatment yielded similar outcomes (Figure S3F–L). Conversely, pharmacological activation of PI3K/AKT signaling with SC79 (AKT activator) enhanced AKT phosphorylation and ZDHHC9 expression in vitro (Figure 3K,L). Administration of SC79 to healthy mice recapitulated DMED‐like phenotypes, including erectile dysfunction and penile structural abnormalities, which were completely abrogated in *Zdhhc9* knockout mice (Figure 3T–Z). Collectively, these findings establish a mechanistic link between PI3K/AKT pathway hyperactivation and ZDHHC9‐driven pathogenesis in DMED (Figure S3M).

### 2.4 ZDHHC9‐Mediated S‐palmitoylation of ACSL4 at the Cys595

**FIGURE 4:**
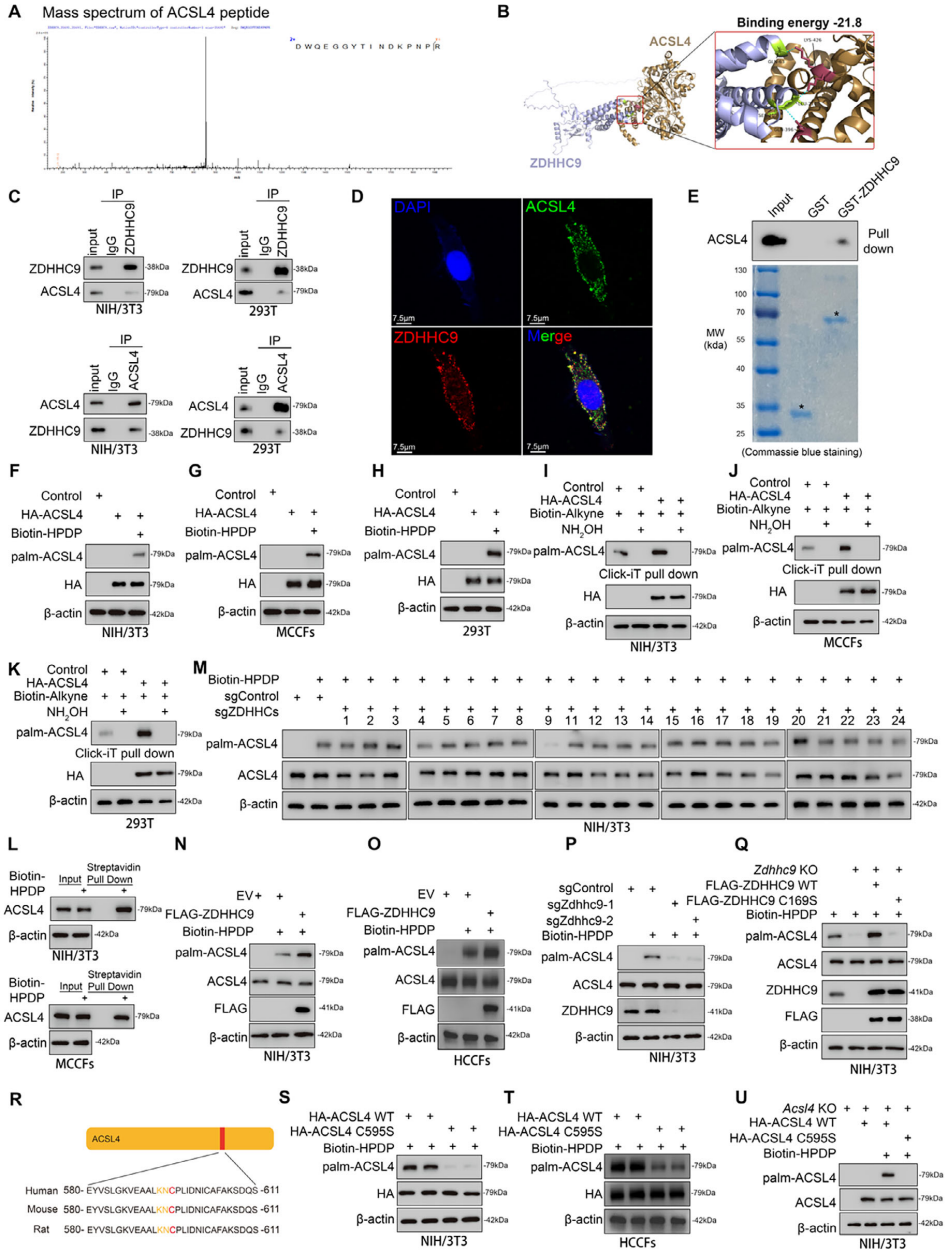
ZDHHC9 mediates palmitoylation of ACSL4 at Cys595. (A) Peptide information of ZDHHC9 and ACSL4 identified by mass spectrometry. (B) Molecular docking between ZDHHC9 and ACSL4 visualized using AlphaFold and PyMOL3. (C) Co‐immunoprecipitation of ZDHHC9 and ACSL4 in 293T and NIH/3T3 cells using specific antibodies (*n* = 3). (D) Confocal microscopy analysis of ZDHHC9 (red) and ACSL4 (green) co‐localization (*n* = 3). Scale bars, 7.5 µm. (E) Western blot analysis of ACSL4 GST pull‐down by recombinant ZDHHC9. (F–H) NIH/3T3, MCCFs, and 293T cells were transfected with empty vector or HA‐ACSL4 plasmids for 72 h, and ACSL4 palmitoylation was detected by ABE assay (*n* = 3). (I–K) NIH/3T3, MCCFs, and 293T cells were transfected with empty vector (EV) or HA‐ACSL4 plasmids for 72 h, and ACSL4 palmitoylation was detected by Click‐iT pull‐down assay (*n* = 3). (L) Endogenous ACSL4 palmitoylation was detected in NIH/3T3 and 293T cells by ABE assay (*n* = 3). (M) NIH/3T3 cells were infected with lentiviruses carrying either a control guide RNA or guide RNAs targeting various ZDHHCs together with Cas9. ACSL4 palmitoylation was evaluated using the ABE assay (*n* = 3). (N,O) NIH/3T3 cells and HCCFs were transfected with empty vector or Flag‐ZDHHC9 plasmids for 72 h, and ACSL4 was labeled with Biotin‐HPDP for streptavidin pull‐down to analyze palmitoylation levels (*n* = 3). (P) Endogenous ZDHHC9 was knocked out in NIH/3T3 cells by CRISPR/Cas9; ZDHHC9‐KO cells were transfected with indicated plasmids for 72 h for immunoblotting analysis, and ACSL4 palmitoylation was assessed by ABE assay (*n* = 3). (Q) NIH/3T3 cells were infected with lentiviruses carrying either a control guide RNA or guide RNAs targeting ZDHHC9 together with Cas9; ACSL4 palmitoylation was analyzed by Biotin‐HPDP streptavidin pull‐down (*n* = 3). (R) Alignment of protein sequences of ACSL4 vertebrate orthologs showing similarity and identity (*n* = 3). (S,T) NIH/3T3 cells and HCCFs were transfected with indicated plasmids for 48 h, and ACSL4 palmitoylation was detected by ABE assay (*n* = 3). (U) Endogenous ACSL4 was knocked out in NIH/3T3 cells by CRISPR/Cas9; ACSL4‐KO cells were transfected with indicated plasmids for 48 h, and ACSL4 palmitoylation was analyzed by ABE assay (*n* = 3). *p*‐values have been indicated in the figures, and *p* < 0.05 is considered statistically significant.

To decipher the molecular mechanism by which ZDHHC9 modulates corpus cavernosum fibroblast homeostasis, we performed immunoprecipitation coupled with microscale proteomic analysis, identifying ACSL4 as a novel interacting partner of ZDHHC9 (Figure 4A). This interaction was corroborated through molecular docking (Figure 4B), co‐immunoprecipitation (Co‐IP; Figure 4C), confocal immunofluorescence co‐localization (Figure 4D), and GST pull‐down assays (Figure 4E), establishing a direct physical association between these proteins.

To determine whether ACSL4 undergoes S‐palmitoylation, we transfected HA‐ACSL4 into NIH/3T3 cells, mouse corpus cavernosum fibroblasts (MCCFs), and HEK293T cells. Palmitoylation was evaluated using the acyl‐biotin exchange (ABE) method with biotin‐HPDP, and streptavidin blotting confirmed that ACSL4 was palmitoylated in all three cell types (Figure 4F–H). Furthermore, Click‐iT labeling with biotin‐alkyne revealed that ACSL4 overexpression elevated global S‐palmitoylation levels, whereas hydroxylamine treatment abrogated this modification, underscoring its dynamic and reversible nature (Figure 4I–K). Endogenous ACSL4 palmitoylation was also detected (Figure 4L), collectively validating ACSL4 as a functionally palmitoylated substrate of ZDHHC9.

To ascertain whether ZDHHC9 directly catalyzes ACSL4 palmitoylation, we generated a CRISPR/Cas9‐mediated knockout panel of individual *Zdhhc* genes in NIH/3T3 cells. Strikingly, ZDHHC9 deletion completely abolished ACSL4 palmitoylation, identifying ZDHHC9 as the predominant palmitoyl acyltransferase for ACSL4 (Figure 4M). Consistent with this, ZDHHC9 overexpression enhanced ACSL4 palmitoylation, whereas its knockout reduced this modification (Figure 4N–P). Notably, a catalytically inactive ZDHHC9 mutant (C169S) failed to rescue palmitoylation defects compared to wild‐type ZDHHC9 (Figure 4Q), highlighting the enzyme's active‐site dependency.

To map the palmitoylation site(s) on ACSL4, we utilized the pCysMod database (http://pcysmod.omicsbio.info/) to predict candidate cysteine residues. Among the candidates, Cys595 exhibited the highest predicted palmitoylation probability and was highly conserved across species (Figure 4R). Substitution of Cys595 with serine (C595S) markedly reduced ACSL4 palmitoylation in NIH/3T3 and human primary corpus cavernosum fibroblast cells (Figure 4S,T). Reintroduction of wild‐type *Acsl4* into *Acsl4*‐KO cells restored palmitoylation, whereas the C595S mutant failed to do so (Figure 4U), confirming Cys595 as the critical site. Taken together, these findings demonstrate that ZDHHC9 directly mediates the palmitoylation of ACSL4 at the conserved Cys595.

### 2.5 ZDHHC9 Promotes Ferroptosis via S‐palmitoylation‐Dependent Regulation of ACSL4

ACSL4 serves as a central node in ferroptosis signaling, orchestrating both initiation and execution of iron‐dependent lipid peroxidation. In vivo, ACSL4 overexpression in murine corpus cavernosum significantly elevated malondialdehyde (MDA) and 4‐hydroxynonenal (4‐HNE) levels—key biomarkers of lipid peroxidation (Figure S4A–C). This was accompanied by significant reductions in ICP, the ratio of interstitial to parenchymal tissue, and the smooth muscle/collagen ratio, establishing a causal link between ACSL4‐driven lipid peroxidation and DMED (Figure S4D–I). Conversely, pharmacological inhibition of ACSL4 enzymatic activity attenuated ferroptosis and improved erectile function in both C57BL/6‐based and db/db mouse models of DMED (Figure S5A–P).

**FIGURE 5:**
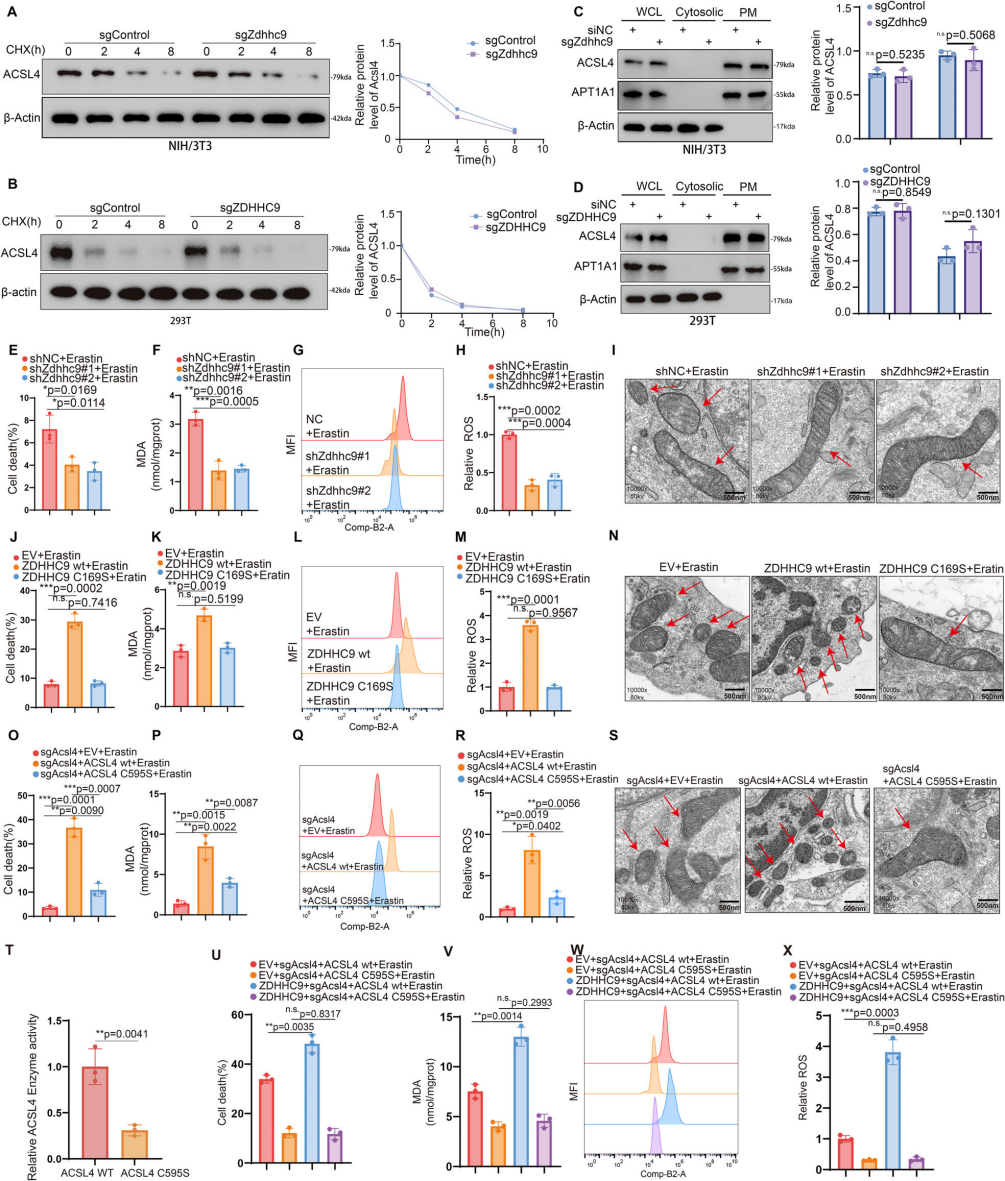
ZDHHC9‐mediated palmitoylation of ACSL4 induces ferroptosis. (A,B) Cycloheximide (50 µg/mL) chase assay; proteins were extracted at 0, 2, 4, and 8 h and analyzed by western blot (*n* = 3). (C,D) Cytosolic and plasma membrane proteins were extracted and analyzed by Western blot (*n* = 3). (E–I) NIH/3T3 cells were infected with indicated shRNAs for 72 h; after puromycin selection, cells were treated with or without Erastin (2 µm) for 24 h, followed by analysis of cell death (E), MDA (F), ROS (G,H), and TEM (I) (*n* = 3). (J–N) NIH/3T3 cells were transfected with indicated plasmids for 24 h, then treated with or without Erastin (2 µm) for 24 h, followed by analysis of cell death (J) (*n* = 3), MDA (K) (*n* = 3), ROS (L,M) (*n* = 3), and TEM (N) (*n* = 3). (O–S) NIH/3T3 cells were treated as indicated, followed by analysis of cell death (O) (*n* = 3), MDA (P) (*n* = 3), ROS (Q,R) (*n* = 3), and TEM (S) (*n* = 3). (T) In vitro ACSL4 enzymatic activity assay (*n* = 3). (U–X) NIH/3T3 cells were treated as indicated, followed by analysis of cell death (U) (*n* = 3), MDA (V) (*n* = 3), and ROS (W,X) (*n* = 3). Bar charts(C,D,E,F,H,J,K,M,O,P,R,T,U,V,X) are presented as mean ± SD. All experiments were performed with at least three biologically independent cell/mouse samples with similar results. Unpaired two‐sided Student's *t*‐test (C,D) and one‐way ANOVA with Tukey's post hoc test (E,F,H,J,K,M,O,P,R,T,U,V,X) were performed. *p*‐values have been indicated in the figures, and *p* < 0.05 is considered statistically significant.

**FIGURE 6:**
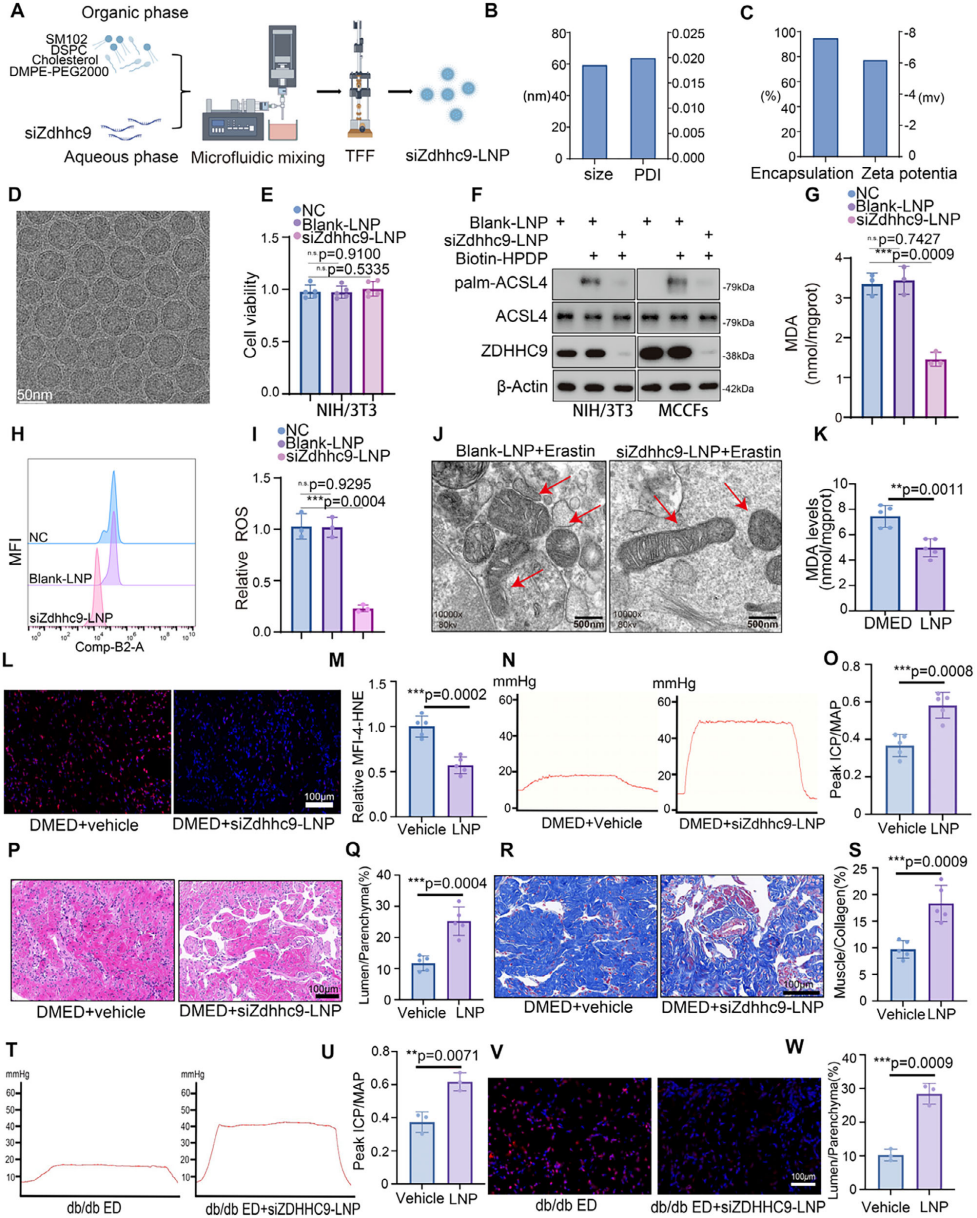
Lipid nanoparticle delivery of Zdhhc9 siRNA rescues DMED in mice. (A) Schematic diagram of LNP synthesis. (B) Average particle size and polydispersity index (PDI). (C) Average encapsulation efficiency and zeta potential. (D) Representative cryo‐EM image showing the morphology of synthesized LNPs. (E) Cell viability in NIH/3T3 cells following LNP treatment (*n* = 5). (F) Western blot analysis of Zdhhc9 expression and ABE assay of ACSL4 palmitoylation in NIH/3T3 and MCCFs after treatment with siZdhhc9‐LNPs. (G–J) NIH/3T3 cells were treated as indicated, followed by analysis of MDA (G) (*n* = 3), ROS (H,I) (*n* = 3), and TEM (J) (*n* = 3). (K) Measurement of MDA levels in the corpus cavernosum (*n* = 5). (L,M) Immunofluorescence staining of 4‐HNE (L) and quantification of MFI (M) (*n* = 5). (N,O) Representative images of ICP and quantification of ICP/MAP ratio (*n* = 5). (P,Q) Morphology and quantification of the corpus cavernosum assessed by H&E staining (*n* = 5). Scale bars, 100 µm. (R,S) Collagen (blue) and smooth muscle (red) evaluated by Masson staining (*n* = 5). Scale bars, 100 µm. (T) Western blot analysis of Zdhhc9 expression and ABE assay of ACSL4 palmitoylation in NIH/3T3 cells following indicated treatments (*n* = 3). (U–W) NIH/3T3 cells were treated as indicated, followed by analysis of MDA (U) (*n* = 3) and ROS (V,W) (*n* = 3). (X,Y) Representative images of ICP and quantification of ICP/MAP ratio (*n* = 3). (Z) Morphology of the corpus cavernosum assessed by H&E staining (*n* = 3). Scale bars, 200 µm. Bar charts(E,G,I,K,M,O,Q,S,U,W,Y) are presented as mean ± SD. All experiments were performed with at least three biologically independent cell/mouse samples with similar results. Unpaired two‐sided Student's *t*‐test (K,M,O,Q,S,U,W,Y) and one‐way ANOVA with Tukey's post hoc test (E,G,I) were performed. *p*‐values have been indicated in the figures, and *p* < 0.05 is considered statistically significant.

Protein palmitoylation is a key post‐translational modification that regulates protein stability, localization, and activation. To investigate whether ZDHHC9 modulates ACSL4 function, we first assessed ACSL4 protein stability using a cycloheximide chase assay. *ZDHHC9* knockout had no effect on ACSL4 degradation (Figure 5A,B). Similarly, subcellular fractionation revealed no changes in ACSL4 localization in the absence of ZDHHC9 (Figure 5C,D). Integrative analysis of publicly available single‐cell RNA‐sequencing datasets from human cavernosal tissue (http://malehealthatlas.cn/) further indicated that ACSL4 expression remained largely unchanged across various cell populations in DMED patients compared with normal controls (Figure 6A). However, functional assays demonstrated that *Zdhhc9* knockdown markedly suppressed Erastin‐induced ferroptosis, as evidenced by reduced cell death (Figure 5E), MDA accumulation (Figure 5F), and intracellular ROS production (Figure 5G,H). Intracellular ferrous ion (Fe^2^^+^) levels were also measured in ZDHHC9 knockdown or ZDHHC9 overexpression cells, with or without treatment with the ferroptosis inducer RSL3. Under basal conditions, no significant differences in Fe^2^^+^ levels were observed among the groups. However, upon RSL3 treatment, ZDHHC9 overexpression markedly exacerbated intracellular iron overload, whereas ZDHHC9 knockdown effectively attenuated RSL3‐induced iron accumulation (Figure S6B,C). In parallel, GPX4 expression remained unchanged under basal conditions regardless of ZDHHC9 expression status. In contrast, following erastin treatment, GPX4 levels were more markedly reduced in ZDHHC9‐overexpressing cells, whereas ZDHHC9 knockdown significantly mitigated erastin‐induced GPX4 downregulation (Figure S6D,E). Transmission electron microscopy revealed that ZDHHC9 deficiency preserved mitochondrial morphology, including cristae integrity and membrane continuity, compared to Erastin‐treated controls (Figure 5I). In vivo, *Zdhhc9* ^ko^ also suppressed ferroptosis in mouse corpus cavernosum tissue without changing the ACSL4 expression (Figure S6F–I). Conversely, *Zdhhc9* overexpression exacerbated ferroptosis, whereas a catalytically inactive mutant (C169S) failed to do so, highlighting the requirement of ZDHHC9's palmitoyltransferase activity (Figure 5J–N).

To define the functional significance of ACSL4 palmitoylation at Cys595, we re‐expressed either wild‐type *Acsl4* or the palmitoylation‐deficient C595S mutant in *Acsl4*‐knockout cells. The C595S mutant exhibited markedly reduced ferroptosis activity compared with wild‐type *Acsl4* (Figure 5O–S). In vitro enzymatic activity assays further demonstrated that the ACSL4 C595S mutant showed a significantly reduced ability to catalyze arachidonoyl‐CoA formation compared with ACSL4 WT (Figure 5T). Moreover, *Zdhhc9* overexpression failed to enhance ferroptosis in cells expressing ACSL4‐C595S (Figure 5U–X). These findings establish ZDHHC9 as a critical upstream regulator of ACSL4‐driven ferroptosis in DMED, with S‐palmitoylation at Cys595 serving as a molecular switch for lipid peroxidation and erectile dysfunction.

### 2.6 Lipid Nanoparticle Delivery of *Zdhhc9* siRNA Ameliorates DMED in Mice

Given the absence of specific pharmacological inhibitors for ZDHHC9, we developed a lipid nanoparticle (LNP) formulation encapsulating siRNA targeting *Zdhhc9* (siZdhhc9‐LNPs) for targeted gene silencing in DMED (Figure 6A). The resulting siZdhhc9‐LNPs exhibited an average particle size of 59.5 nm, a polydispersity index (PDI) of 0.02, a zeta potential of −6.2 mV, and an encapsulation efficiency of 95% (Figure 6B,C). Cryo‐electron microscopy confirmed the uniform morphology and nanoscale structure of the particles (Figure 6D). Cytotoxicity assays revealed minimal toxicity (Figure 6E). Fluorescence analysis of labeled siZdhhc9‐LNPs indicated efficient local distribution in the corpus cavernosum at multiple time points after intracavernosal injection (Figure S7A–C). Functionally, siZdhhc9‐LNPs effectively silenced ZDHHC9 expression, accompanied by reduced ACSL4 palmitoylation (Figure 6F). Under erastin‐induced ferroptosis, siZdhhc9‐LNPs significantly attenuated lipid peroxidation and ROS accumulation (Figure 6G–I). Transmission electron microscopy further revealed preserved mitochondrial cristae and membrane integrity in treated cells (Figure 6J), corroborating the protective effects against ferroptosis damage. In vivo, administration of siZdhhc9‐LNPs in DMED mice markedly decreased corpus cavernosum levels of MDA and 4‐HNE (Figure 6K–M). Functional assessments demonstrated significant improvements in erectile function, including elevated ICP, enhanced trabecular/sinusoidal ratio, and restored smooth muscle/collagen balance—mirroring the therapeutic outcomes observed in the type 2 diabetic mouse model of erectile dysfunction (Figure 6N–W). Concurrently, *Zdhhc9* knockdown effectively reduced extracellular collagen production (Figure S7D). Importantly, LNP treatment did not induce hepatotoxicity or nephrotoxicity, as assessed by serum biochemical analysis (Figure S7E–H). Target specificity was rigorously validated using *Zdhhc9* knockout models. In *Zdhhc9*‐deficient cells, siZdhhc9‐LNPs failed to further suppress ACSL4 palmitoylation (Figure S8A) or reduce MDA and ROS levels (Figure S8B–D). Similarly, *Zdhhc9* ^ko^ mice exhibited no therapeutic response to LNP treatment, with unaltered ICP, trabecular/sinusoidal ratio, and extracellular matrix composition (Figure S8E–J). These findings confirm that the antiferroptosis and functional benefits of siZdhhc9‐LNPs are exclusively mediated through ZDHHC9 inhibition.

Together, these findings demonstrate that siZdhhc9‐LNPs precisely target *Zdhhc9*, suppress downstream palmitoylation of ACSL4, and exert therapeutic efficacy in DMED, offering a promising strategy for DMED treatment.

## 3 Discussion

**FIGURE 7:**
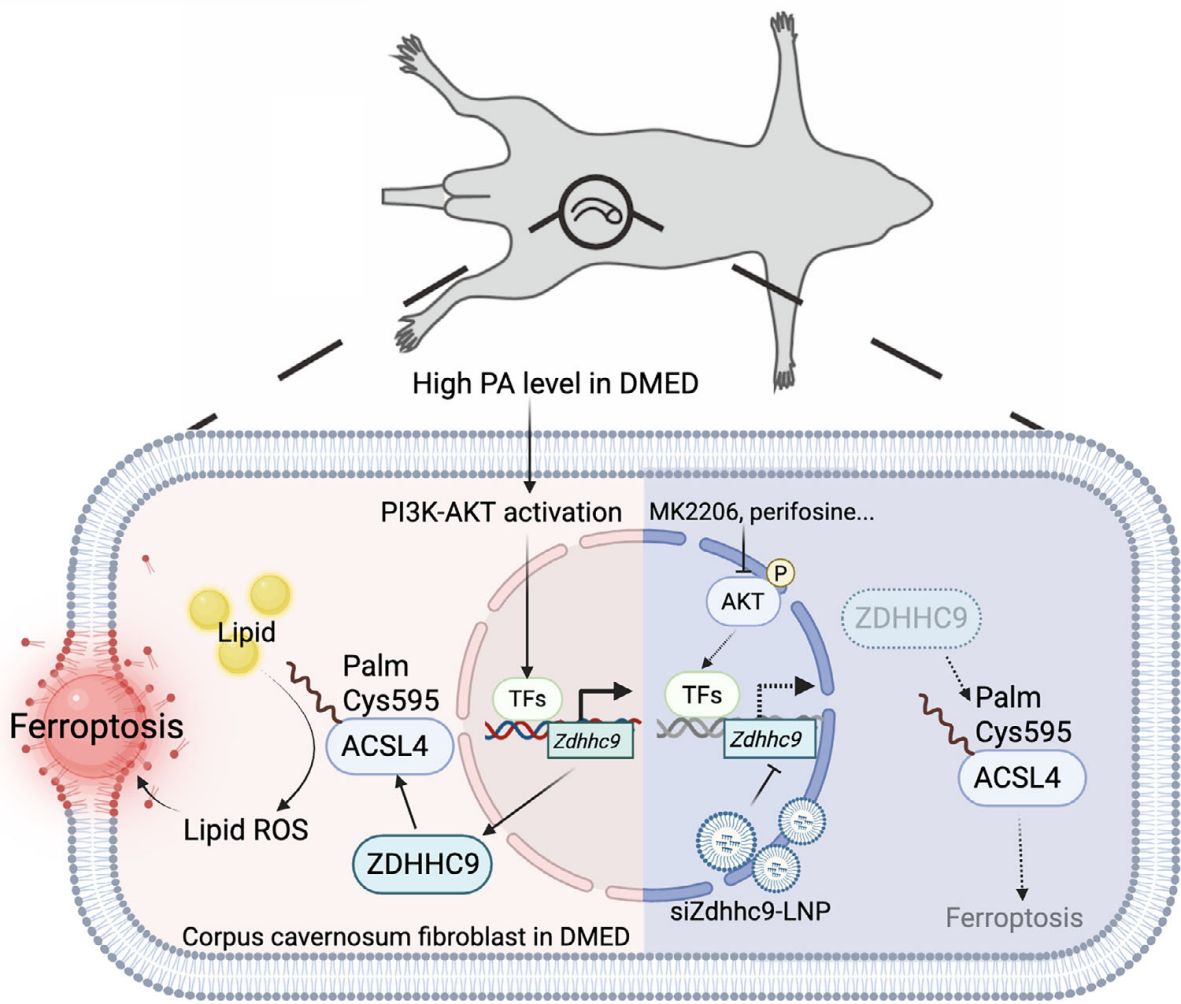
Metabolic dysregulation in DMED promotes ferroptosis in corpus cavernosum fibroblasts via a ZDHHC9‐ACSL4 axis. Elevated PA levels in DMED activate the PI3K/AKT pathway, leading to aberrant upregulation of the palmitoyltransferase ZDHHC9. ZDHHC9 catalyzes S‐palmitoylation of ACSL4 at cysteine 595 (Cys595). This post‐translational modification amplifies lipid peroxidation, triggering iron‐dependent ferroptosis in penile fibroblasts. Therapeutic targeting of ZDHHC9 using lipid nanoparticle‐encapsulated siRNA (siZdhhc9‐LNPs) suppresses ACSL4 palmitoylation and ferroptosis, restoring corpus cavernosum integrity and erectile function in DMED mice. Mechanistic schematic highlights the metabolic‐ferroptosis crosstalk as a druggable pathway for DMED. Created in BioRender.com.

Using untargeted metabolomics, we identified a significant elevation of PA in the serum of patients with DMED. Consistently, quantification of palmitic acid in the corpus cavernosum tissue of mice demonstrated a marked elevation in the DMED group relative to the control group. Notably, injection of 2‐BP, a palmitoylation inhibitor, effectively improved erectile function in DMED mice. Transcriptomic profiling of rat and mouse corpus cavernosum, along with human single‐cell RNA‐seq data, revealed a marked upregulation of *ZDHHC9* in cavernosal fibroblasts under DMED conditions. Mechanistically, aberrant activation of the PI3K/AKT signaling pathway in DMED was identified as a primary driver of ZDHHC9 upregulation. ZDHHC9 catalyzes the palmitoylation of ACSL4, enhancing its enzymatic activity and promoting lipid peroxidation, ultimately leading to ferroptosis in cavernosal fibroblasts. Therapeutic delivery of siZdhhc9 via LNPs into the corpus cavernosum markedly improved erectile function in DMED mice, underscoring ZDHHC9 as a promising therapeutic target for DMED (Figure 7).

Metabolic disorder is a major contributing factor to the development of various diabetic complications. Numerous studies have demonstrated that lipid metabolic disorder plays a critical role in driving multiple diabetes‐related pathological processes [25]. In particular, elevated triglycerides, increased low‐density lipoprotein (LDL), and reduced high‐density lipoprotein (HDL) levels in diabetic patients have been identified as significant risk factors for atherosclerosis [26]. Recent studies have shown elevated serum palmitic acid levels in individuals with type 2 diabetes mellitus, which are closely associated with unstable coronary atherosclerotic plaques [27]. Our findings further confirm that PA levels are significantly increased in DMED patients, and prolonged exposure to high concentrations of PA impairs erectile function in mice.

As a highly abundant free fatty acid in humans, PA is converted to palmitoyl‐CoA and subsequently transferred onto specific amino acid residues of proteins by palmitoyltransferases, resulting in palmitoylation [23]. This lipid post‐translational modification plays critical roles in various diseases by regulating protein stability, localization, and activity. In metabolic disorders, aberrant palmitoylation of insulin receptors disrupts insulin signaling and contributes to insulin resistance [28]. In the nervous system, neuronal activity and plasticity are accompanied by dynamic changes in protein palmitoylation, and perturbation of palmitoylation/depalmitoylation disrupts synaptic transmission and plasticity [29]. In cancer, aberrant palmitoylation enhances protein activity to promote tumor cell proliferation, migration, and invasion. For instance, PD‐L1 palmitoylation stabilizes the protein by preventing its ubiquitin‐mediated lysosomal degradation, thereby promoting immune evasion [30].

Protein palmitoylation is catalyzed by 23 members of the ZDHHC family [31]. To identify the key palmitoyltransferase involved in DMED, we integrated transcriptomic data from rat and mouse corpus cavernosum and human single‐cell sequencing, identifying *ZDHHC9* as significantly upregulated in DMED cavernosal fibroblasts. Genetic deletion of *Zdhhc9* restored erectile function and reduced fibrosis in DMED mice. ZDHHC9, a zinc finger‐containing DHHC family member, regulates various physiological processes such as cell proliferation, differentiation, and signaling through palmitoylation, and its dysregulation is implicated in metabolic diseases and cancer. In T2DM‐associated bone metabolic disorders, high‐glucose environments upregulate ZDHHC9 in osteoblasts, suppressing osteogenic differentiation through palmitoylation. ZDHHC9 knockout improves bone regeneration around implants in diabetic mice, underscoring its promise as a target for metabolic bone disorders [32]. In pancreatic cancer models, silencing ZDHHC9 reshapes the immunosuppressive tumor microenvironment into a pro‐inflammatory one, thereby inhibiting tumor progression and prolonging survival [33]. Lipid metabolic disorder in DMED can also activate key signaling pathways such as PI3K/AKT, which orchestrates glucose uptake, glycogen synthesis, and lipid metabolism [34]. Our findings demonstrate that lipid metabolism disorders contribute to PI3K‐AKT pathway dysregulation, which correlates with ZDHHC9 upregulation in DMED. While the molecular mechanisms governing this regulatory axis require further investigation, these results highlight a previously unrecognized pathogenic link between metabolic‐ferroptosis crosstalk and penile fibroblast dysfunction.

Ferroptosis, a regulated form of cell death driven by lipid peroxidation, is tightly controlled by enzymes such as GPX4 and ACSL4 [35]. GPX4 suppresses ferroptosis by reducing lipid peroxides in a GSH‐dependent manner, whereas ACSL4 promotes it by generating acyl‐CoA substrates for phospholipid peroxidation and facilitating lipid peroxide accumulation [7]. Together, these enzymes form a core regulatory network that maintains the dynamic balance of lipid peroxidation. In DMED, ferroptosis is a key pathological contributor, and the ACSL4/LPCAT3/LOX axis has been implicated in iron overload, oxidative stress, and fibrosis [12]. However, the upstream regulatory mechanisms of these ferroptosis‐related factors remain poorly understood.

Recent studies have shown that protein S‐palmitoylation can modulate ferroptosis, with most attention focused on GPX4. For example, Yin et al. demonstrated that palmitoylation of GPX4 at Cys66 by ZDHHC20 enhances its stability, while APT2‐mediated depalmitoylation reduces it, thereby altering ferroptosis sensitivity [20]. Zhou et al. reported that GPX4 is palmitoylated at Cys75 by ZDHHC8, which increases its stability; pharmacological inhibition of ZDHHC8 with PF‐670462 sensitizes tumor cells to ferroptosis and promotes antitumor immunity by reducing GPX4 palmitoylation [21]. In contrast, whether palmitoylation regulates ACSL4—a key driver of lipid peroxide generation—has not been explored. Here, we identify ZDHHC9‐mediated palmitoylation of ACSL4 at Cys595 as a potent inducer of ferroptosis. Notably, our experiments showed that manipulation of ZDHHC9 alone did not induce marked changes in intracellular iron accumulation or GPX4 expression under basal conditions, whereas these effects became pronounced in the presence of ferroptosis inducers. This observation is highly consistent with our proposed mechanism that ZDHHC9 promotes ferroptosis primarily through palmitoylation‐dependent activation of ACSL4. As a key executor of ferroptosis, ACSL4 determines the availability of polyunsaturated fatty acid‐containing phospholipid substrates for lipid peroxidation, thereby influencing cellular susceptibility to ferroptotic stress rather than acting as a standalone trigger of cell death. In this context, ZDHHC9‐mediated palmitoylation of ACSL4 appears to function as a molecular amplifier that enhances ferroptosis responsiveness under metabolic or pharmacological stress conditions, rather than being sufficient on its own to induce overt ferroptosis in the absence of such stimuli.

Previous studies have shown that palmitoylation can influence protein stability and subcellular localization [36]. However, our protein half‐life and membrane fractionation experiments indicate that ZDHHC9‐mediated palmitoylation of ACSL4 at Cys595 does not affect its stability or membrane localization. Moreover, palmitoylation has also been reported to regulate enzymatic activity [37]. Cys595 is located within the catalytic core of ACSL4, which contains a conserved acyl‐CoA synthetase triad (Lys–Asp–Cys) [38]. In vitro, S‐palmitoylation of ACSL4 at Cys595 altered MDA and ROS levels and modulated ferroptosis‐related phenotypes, indicating that palmitoylation at this site regulates ACSL4 function. Collectively, our findings identify ZDHHC9‐mediated palmitoylation of ACSL4 at Cys595 as a critical molecular switch that activates its enzymatic function without altering protein stability or localization, revealing a previously unrecognized palmitoylation‐dependent mechanism controlling lipid peroxide production in ferroptosis and filling a key gap in the field.

To date, no potent and specific inhibitors of ZDHHC family enzymes are available. Although 2‐BP has been identified as a broad‐spectrum palmitoylation inhibitor through high‐throughput screening, its non‐selectivity and off‐target effects limit its clinical utility [39]. Small interfering RNAs (siRNAs) have recently emerged as a promising therapeutic strategy for diabetes‐related diseases [40, 41, 42]. However, clinical application of siRNA therapy remains challenging due to issues such as poor stability, low cellular uptake, and off‐target effects. Lipid nanoparticles (LNPs) composed of ionizable lipids, PEGylated lipids, and cholesterol provide a nanoscale delivery platform that protects siRNA, enhances cellular uptake, and enables targeted delivery. LNPs have shown clinical success in various therapeutic contexts [43, 44, 45]. In our study, intra‐cavernosal injection of siZdhhc9‐LNP effectively silenced ZDHHC9 expression and significantly improved erectile function and fibrosis in DMED mice.

Despite these promising findings, several limitations warrant further investigation. First, our mechanistic studies were primarily conducted in a single STZ‐induced DMED mouse model, which may not fully recapitulate the clinical heterogeneity of human T2DM‐associated ED. In addition, the lack of experiments using primary fibroblasts from DMED patients constrains the clinical relevance of the study. Second, although our study focuses on ACSL4 palmitoylation, we cannot exclude the possibility that ZDHHC9 may regulate cavernosal fibroblast function through ACSL4‐independent mechanisms. Third, while the siZdhhc9‐LNP system demonstrated short‐term efficacy and safety, its long‐term biocompatibility, stability, targeting specificity, and optimal delivery route require further evaluation through comprehensive analyses.

In summary, this study integrates metabolomics, transcriptomics, and functional assays to reveal a novel pathogenic axis—“PI3K/AKT/ZDHHC9/ACSL4/ferroptosis”—underlying DMED. We further validate the therapeutic potential of targeting Zdhhc9 using an siZdhhc9‐LNP delivery system. While challenges remain, our findings provide new mechanistic insights into DMED pathogenesis and highlight ZDHHC9 as a compelling therapeutic target. Future studies focusing on multi‐model validation, comprehensive substrate mapping, and delivery system optimization are warranted to facilitate translational advancement.

## 4 Experimental Section

### 4.1 Cell Culture

The NIH/3T3 cell line, mouse embryonic fibroblasts, was obtained from Cas9x(China). The HEK293T cell line was obtained from Cas9x(China). Human primary corpus cavernosum fibroblasts were obtained from JieXinBio(China). Primary mouse corpus cavernosum fibroblasts were isolated as previously reported [46]. In brief, the penis of an 8‐week‐old C57BL/6 mouse was obtained. The glans penis, urethra, dorsal blood vessels, and nerves were removed, then tissues were cut into 1‐mm^3^ volumepieces; the tissues were immersed in 15 mL PBS with vigorous shaking to remove residual blood cells. Then, tissues were cut into pieces of 1 mm × 1 mm size and enzymatically digested with 2.5 mg/mL collagenase type I (#17100017, thermofisher), 4 mg/mL collagenase type IV (#17104019, thermofisher), and 0.1 mg/mL neutral protease(#HY‐131577A, MCE) for 40 min. The digested single‐cell suspensions were washed and resuspended in 10% FBS/DMEM (Gibco).

### 4.2 Reagents

Apomorphine and streptozotocin were purchased from Sigma–Aldrich (MO, USA). The following antibodies were used for western blotting analysis: SLC1a3(# 20785‐1‐AP, Proteintech, 1:2000), ZDHHC9(ab74504, abcam, 1:1000), AKT(Ser473)(#66444‐1‐Ig, Proteintech, 1:2000), AKT(10176‐2‐AP, Proteintech, 1:2000), β‐actin (#20536‐1‐AP, Proteintech, 1:5000), ACSL4(#22401‐1‐AP, Proteintech, 1:5000), KLF4(ab215036, abcam, 1:1000), SOX2(ab97959, abcam, 1:1000), HIF1α(#20960‐1‐AP, Proteintech, 1:2000), PRRX1(#HPA051084, sigma), FOXA1(#17‐10267, sigma), Flag tag(#AE092, ABclonal, 1:5000), HA tag(#51064‐2‐AP, Proteintech, 1:5000); For immunofluorescence(IF) of mouse tissue, the following antibody were used: Slc1a3(#20785‐1‐AP, Proteintech, 1:200), CD31(#11265‐1‐A, Proteintech, 1:100), ZDHHC9(#PA5‐56868, Thermofisher, 1:50), ACSL4(#MA5‐49273, Thermofisher, 1:100), 4‐HNE(#MA5‐27570, Thermofisher, 1:100); 2‐BP(#E0120), SC79(S7863), Palmitic acid(S3794), MK2206(S1078) and Perifosine (S1037) were purchased from Selleckchem. AAV were provided by Ji‐kai Biotechnology (Shanghai, China); The cDNA templates of Acsl4, ACSL4, Zdhhc9, and ZDHHC9 were acquired from Miaolingbio (Wuhan, China). They were truncated with routing protocols. The full‐length and truncated forms were subcloned in the backbone plasmid, including PCDH‐3×Flag, and PCDH‐3×HA. For point mutation, mutated sites were designed in the primers, and the cDNA sequence was divided into two parts by the PCR procedure. Subsequently, fusion PCR was performed to obtain the mutated full‐length cDNA sequence.

The siRNAs were obtained from RiboBio. The sequences of the siRNAs and single‐guide RNAs (sgRNA) are provided in Table S1. Cells were transfected with the plasmids or siRNAs using Lipofectamine 2000 (Thermo Fisher Scientific). The sources of other reagents are clearly noted in each part.

### 4.3 Transfection of siRNA and Plasmids

The designated siRNA, plasmids, and Lipofectamine 2000 (Thermo Fisher Scientific) were mixed in 1 mL serum‐free Opti‐MEM. After 20 min, the mixture was added to the cells starved for 12 h. After 6 h of transfection, the culture medium was replaced with complete DMEM medium for another 72 h.

### 4.4 Co‐Immunoprecipitation and Western Blot

Cells were collected and lysed using RIPA lysis buffer (#G2033, servicebio, China). Then, 30 µL of protein A+G beads (#L‐1004, bio‐linkedin, China) were added to the lysate, along with either IgG (#30000‐0‐AP, proteintech, China) or a primary antibody. After incubating for over 24 h, the beads were washed with RIPA buffer. For Western blotting, the protein lysates were first boiled and then separated by SDS‐PAGE. After electrophoresis, the proteins were transferred onto a nitrocellulose membrane. Protein bands were visualized using an Multifunctional imaging system (SCG‐W5000, servicebio, China).

### 4.5 RT‐qPCR

RNA was extracted with TRIzol (Thermo Fisher Scientific) and reverse‐transcribed with Prime Script RT Kit (Takara). The cDNA was amplified using TB Green Fast qPCR Mix (Takara). Relative gene expression was determined by the 2^−ΔΔCt^ method after normalization to β‐actin. The sequences of primers are provided in Table S2.

### 4.6 Luciferase Assay

Plasmids containing the ZDHHC9 promoter were synthesized by Miaolingbio (Wuhan, China). These constructs were co‐transfected into 293T cells together with either an empty vector (EV) plasmid or an overexpression plasmid for HIF1A, SOX2, PRRX1, KLF4, or FOXA1. After 48 h, the cells were collected, and luciferase activity was quantified using the Dual‐Luciferase Reporter Assay System (Promega) following the manufacturer's protocol. Firefly luciferase signals were normalized to Renilla luciferase to account for transfection efficiency, and results are expressed as the firefly/Renilla activity ratio.

### 4.7 Coimmunoprecipitation and Liquid Chromatography Coupled to Tandem Mass Spectrometry

Total proteins were extracted using IP buffer and incubated with protein A+G beads (#L‐1004, bio‐linkedin, China) and IgG (#30000‐0‐AP, proteintech, China) or primary antibody for 24 h. The beads were collected and washed with PBS three times. After boiling with an SDS‐loading buffer for 10 min, the eluted protein was analyzed by gel electrophoresis and liquid chromatography–tandem mass spectrometry (LC–MS/MS).

### 4.8 Chromatin Immunoprecipitation (ChIP) and ChIP‐qPCR

ChIP were carried out following the instructions of the Chromatin Extraction Kit (Abcam) and the ChIP Kit Magnetic‐One‐Step Method (Abcam). The purified DNA was analyzed with the identical approach used for cDNA in RT‐qPCR. The primer sequences for ChIP—qPCR are listed in Table S3.

### 4.9 Glutathione S‐transferase (GST) Pulldown Assay

As previously described [47], cells were lysed with 1 × RIPA lysis buffer (#G2033, servicebio, China) for 30 min at 4°C. Glutathione S‐transferase (GST) fusion proteins were immobilized on Anti‐GST Magnetic Beads (L‐1014, bio‐linkedin). After washing with 1 × RIPA lysis buffer, the beads were incubated with cell lysates for 4 h. The beads were then washed four times with 1 × RIPA lysis buffer and resuspended in loading buffer. The bound proteins were subjected to SDS/PAGE and Western blotting.

Escherichia coli BL21 was used to express GST‐recombinant proteins after induction with IPTG (ST098, Beyotime). Then Escherichia coli BL21 was lysed with muramidase and sonication. Bacterial debris was removed by centrifugation for 10 min. Glutathione‐Sepharose beads (L‐2004, bio‐linkedin) were added to the liquid supernatant to purify GST‐fusion proteins at 4°C overnight. The beads were then collected and washed for six times with binding buffer to remove the contaminating proteins. SDS‐PAGE and high‐sensitivity colloidal Coomassie blue staining were performed to examine the purification efficiency.

### 4.10 Acyl‐biotinyl Exchange Assay

Cell lysates were incubated with 20 mmol/L of methyl methanethiosulfonate (Sigma–Aldrich) and 1 mmol/L PMSF (Beyotime, China) at 50°C for 30 min to exhaustively block free thiols. Proteins were precipitated with acetone and resuspended in 1 mol/L hydroxylamine pH 7.4 (Sigma–Aldrich) to promote depalmitoylation. The proteins were incubated with 0.2 mmol/L biotin‐HPDP (Top Science) for 1 h at room temperature. Streptavidin (Yeasen) was used to purify biotinylated proteins, and biotinylated proteins were analyzed by immunoblotting.

### 4.11 Click‐iT Pull Down

For click‐iT identification of palmitoylation, click chemistry and streptavidin pulldown were performed according to the published procedure with slight modifications [48]. NIH/3T3, MCCFs, and HEK293T cells were transfected with the indicated plasmids for 48 h. Subsequently, the cells were incubated with 100 µmol/L of Click‐iT palmitic acid‐azide (Thermo Fisher Scientific) for 6 h. After incubation, the cells were lysed to extract proteins. A Click‐iT Protein Reaction Buffer Kit (# C10276; Thermo Fisher Scientific) was used to catalyze the reaction of protein samples with biotin‐alkyne. The biotin alkyne‐azide–palmitic‐protein complex was precipitated with streptavidin agarose beads (Biolinkedin, China), and the bound proteins were eluted by boiling with SDS‐PAGE sample buffer without DTT for 10 min at 95°C, and then analyzed through immunoblotting.

### 4.12 Protein Purification

293T (ACSL4 knockout) cells were maintained in DMEM supplemented with 10% fetal bovine serum at 37°C in a humidified atmosphere containing 5% CO_2_. Cells were transfected at approximately 70%–80% confluence using Lipofectamine 3000 with GST‐ACSL4 wild‐type (WT), GST‐ACSL4 C595S mutant, or GST empty vector plasmids (10 µg per 10‐cm dish). At 48 h post‐transfection, cells were harvested and lysed on ice in NP‐40 lysis buffer (50 mm Tris‐HCl (pH 7.4), 150 mm NaCl, 0.5% NP‐40, 10% glycerol, 1 mm DTT, and EDTA‐free protease inhibitors). Lysates were clarified by centrifugation at 12 000 g for 10 min at 4°C, and the supernatants were incubated with pre‐equilibrated GST magnetic agarose beads at 4°C for 1 h with gentle rotation. The beads were washed multiple times to remove nonspecifically bound proteins, and GST fusion proteins were eluted using glutathione‐containing elution buffer. Eluted proteins were pooled and subjected to buffer exchange using PD‐10 desalting columns equilibrated with reaction buffer. Protein concentrations were determined by the BCA assay, and protein purity was assessed by SDS–PAGE followed by western blotting using an anti‐GST antibody.

### 4.13 In Vitro Enzymatic Assay

Purified and buffer‐exchanged GST‐ACSL4 proteins were subjected to in vitro enzymatic assays at 37°C in a total reaction volume of 50 µL containing 50 mm Tris‐HCl (pH 7.4), 10 mm MgCl_2_, 1 mm DTT, 0.1% BSA, 300 µm CoA, 2 mm ATP, and 100 µm arachidonic acid. Reactions were initiated by the addition of GST‐ACSL4 (0.1 µg per reaction) and incubated at 37°C with shaking. Reactions were quenched with ice‐cold methanol, and the supernatants were collected after centrifugation at 12 000 g for 10 min at 4°C. Samples were purified using C18 solid‐phase extraction, dried, and reconstituted for LC–MS analysis. Enzymatic activity was determined based on arachidonoyl‐CoA formation and expressed as relative values normalized to ACSL4 WT. Negative controls, including GST alone and reactions lacking ATP or CoA, were included in each experiment.

### 4.14 Intracellular Reactive Oxygen Species (ROS) Detection

For ROS detection, A ROS detection kit (#S0033S, Beyotime, China) with the fluorescent probe DCFH‐DA was used. DCFH‐DA was diluted 1:1000 with serum‐free medium to a final concentration of 10 µm/L. The cells were suspended in the diluted DCFH‐DA and incubated in a cell culture incubator at 37°C for 20 min. After washing with PBS three times, the cells were resuspended in 200 µL PBS and analyzed by flow cytometry (Beckman Coulter, CA, USA).

### 4.15 MDA Detection

For MDA detection, a cellular MDA assay kit (#S0131S, Beyotime, China) was used employing the thiobarbituric acid method. And the MDA level was adjusted based on the cell protein concentration.

### 4.16 Transmission Electron Microscopy (TEM)

Cells were fixed with electron microscope fixative (Servicebio, China), then washed in 0.1 m sodium cacodylate buffer and treated with 0.1% Millipore‐filtered cacodylate‐buffered tannic acid, postfixed with 1% buffered osmium, and stained en bloc with 1% Millipore‐filtered uranyl acetate. The samples were dehydrated in increasing concentrations of ethanol, infiltrated, and embedded. The samples were polymerized in a 60°C oven for approximately 3 days. Ultrathin sections were cut using an ultramicrotome, stained with uranyl acetate and lead citrate in a stainer. Finally, the ultrathin sections were examined by transmission electron microscopy to analyze the morphological changes of mitochondria.

### 4.17 Cell Counting Kit‐8 (CCK‐8) Assay

CCK‐8 assay was performed by CCK‐8 kit (#C0037, Beyotime, China). Cells from different treatment groups were collected, thoroughly resuspended in culture medium, and counted. The cell suspension was then diluted to ensure that each well of the 96‐well plate contained 5000 cells in 100 µL (adjusted based on cell viability). Each group included at least three replicates. Wells containing only culture medium (without cells) were reserved as blank controls.CCK‐8 solution was added to each well and incubated for 1 h. The absorbance of each well was measured at a wavelength of 450 nm using a microplate reader to assess cell viability.

### 4.18 CRISPR Technique

Multiple sgRNAs targeting Zdhhcs and ACSL4 were designed using the platform available at https://www.synthego.com. Subsequently, these sgRNAs were cloned into the lentiCRISPR v2 vector (Addgene). The sequences of the sgRNAs are presented in Table S1.

### 4.19 LNP

siZdhhc9 was purchased from RiboBio. To prepare the lipid nanoparticle (LNP) formulation, an aqueous phase containing siRNA at a concentration of 0.35 mg/mL was prepared using 100 mm sodium acetate buffer (pH 4.0). Simultaneously, an organic phase was prepared by dissolving lipids in absolute ethanol at a total lipid concentration of 25 mm. The lipid components were as follows: SM102, Cholesterol, DSPC, DMG‐PEG 2000. The aqueous and organic phases were mixed at a volume ratio of 3:1 with a total flow rate of 12 mL/min using an Ignite microfluidic chip on a PNI Ignite instrument to encapsulate the siRNA into LNPs. The hydrodynamic diameter, zeta potential, and polydispersity index (PDI) of the LNPs were measured by Zetasizer Nano ZS. The morphology of LNPs was further examined by Cryo‐electron microscopy.

To evaluate the local distribution of LNPs in the corpus cavernosum, Cy5‐labeled siZdhhc9‐LNPs were administered via intracavernosal injection to healthy C57BL/6 mice. Corpus cavernosum tissues were harvested at the indicated time points, embedded, sectioned, and subjected to fluorescence imaging or multiplex immunofluorescence staining to assess the distribution of Cy5‐labeled LNPs in the tissue.

To evaluate the biodistribution of LNPs [49], DiD‐labeled LNPs encapsulating siZdhhc9 were administered via intracavernosal injection to healthy C57BL/6 mice. Tissues—specifically the corpus cavernosum, liver, skeletal muscle, spleen, and brain—were harvested at defined intervals (0.25, 1, 4, and 12 h post‐injection), weighed, and homogenized in PBS. The fluorescence intensity of the tissue homogenates was then measured using a multimode microplate reader to compare the relative distribution of LNPs among different tissues.

### 4.20 PM Protein and Cytosolic Proteins Extraction Assay

Cell membrane protein was collected by using a plasma membrane protein extraction kit (Abcam, ab65400). In brief, cells were incubated with 2 mL of homogenized buffer at 4°C after being washed three times with PBS buffer. The cells were then harvested with a cell scraper. Following this process, the cells were homogenized with a homogenizer for 50 times and spun at 700 × g for 5 min. Then the supernatants were collected and centrifuged at 10 000 × g for 30 min at 4°C. Total cellular membrane proteins and cytosolic proteins were located in the pellet and supernatant, respectively.

### 4.21 Animals

All animal experiments were approved by the Animal Use and Care Committee of the Second Xiangya Hospital, Central South University (Approval No. 20250778), and conducted in accordance with the Declaration of Helsinki.

The DMED model was induced using a combination of a high‐fat diet and streptozotocin (STZ) injection, as previously described [50]. The high‐fat diet was purchased from Medison Biotechnology Co., Ltd. with the product number d12492. For STZ preparation, citrate buffer was first formulated: Solution A was prepared by dissolving 2.1 g citric acid in 100 mL double‐distilled water; Solution B was prepared by dissolving 2.94 g sodium citrate in 100 mL double‐distilled water; then Solution A and Solution B were mixed at a 1:1 ratio to obtain the citrate buffer for STZ dissolution.

Male C57BL/6 mice underwent a 2‐week acclimatization period before being randomly assigned to control or experimental groups. Mice in the experimental group were fed the aforementioned high‐fat diet for 4 consecutive weeks, followed by intraperitoneal injections of STZ (freshly dissolved in the prepared citrate buffer, pH ∼4.5) at a dose of 50 mg/kg/day for 5 consecutive days. Diabetes was confirmed only when blood glucose levels exceeded 16.7 mmol/L on three consecutive measurements. These diabetic mice then continued on a high‐fat diet for another 8 weeks to induce DMED. To assess erectile function, apomorphine was dissolved in 0.9% saline containing 0.2 mg/mL ascorbic acid and administered subcutaneously at a dose of 100 µg/kg. Erectile responses were visually monitored for 30 min post‐injection. Mice that failed to display penile erection during this period were classified as successful DMED models. Out of 200 initially enrolled mice, 35 died due to severe hyperglycemia or procedural complications, leaving 165 diabetic survivors. Among these, 147 met the DMED criteria (no erectile response) and were randomly assigned to treatment subgroups. The success rate of DMED modeling was 89.1% (147/165), demonstrating the efficiency and reliability of this method. All male C57BL/6 mice in both the control and DMED groups were strictly age‐matched to minimize potential confounding effects of age on the experimental outcomes.

DMED mice were then randomly divided into ten groups: the 2‐BP group received intraperitoneal injections of 2‐BP (10 mg/kg), with 5% DMSO in corn oil used as the vehicle control; the MK2206 group was administered MK2206 by oral gavage at 480 mg/kg once per week, with saline as the control; the perifosine group received daily oral gavage at 36 mg/kg, with saline‐treated mice as controls; the siZdhhc9‐LNP group received intracavernosal injections of siZdhhc9‐loaded lipid nanoparticles (0.25 mg/mL), with blank LNPs serving as controls; the PRGL493 group received intraperitoneal injections of PRGL493(1µg/kg), with 5%DMSO and 95% oil as controls. Four weeks post‐treatment, cavernous ZDHHC9 expression, intracavernosal pressure (ICP), and histopathological changes were assessed.

Additionally, normal male C57BL/6 mice were randomly divided into six groups for mechanistic investigation: the PA group received intraperitoneal injections of palmitic acid (10 mg/kg), with BSA‐treated mice as controls; the SC79 group was treated intraperitoneally with SC79 (10 mg/kg) twice per week, with vehicle‐treated mice as controls; ACSL4 overexpression mice were administered AAV‐ACSL4^oe^ (3 × 10^1^ ^3^ vg/mL) via intracavernosal injection, with AAV‐empty vector as the control.

Zdhhc9^ko^ mice were generated by Shulaibao Biotechnology (Wuhan, China). Age‐matched male wild‐type C57BL/6 mice served as controls. The DMED model in Zdhhc9^ko^ mice was established following the same induction protocol. After successful modeling, cavernosal ZDHHC9 expression, ICP, and histopathological alterations were evaluated across four groups: normal, Zdhhc9^ko^, DMED, and Zdhhc9^ko^ ‐DMED.

Additionally, 20‐week‐old male *db*/*db* and *db*/*m* mice (C57BLKS/J‐leprdb/leprdb) were obtained from Shulaibao Biotechnology (Wuhan, China). The DMED model in *db*/*db* mice was established following the same protocol. ICP and histopathological alterations were evaluated across the different groups receiving various treatments.

### 4.22 ICP

Combined with the literature [51] and previous experience, we used the BL‐420F Functional Biological Experiment System (China) to measure the ICP. After anesthesia with isoflurane, the crura of the penis on both sides and the cavernous nerves were exposed. A 24G needle was inserted into the crus of the penis on one side. Meanwhile, a bipolar hook‐shaped electrode was used to stimulate the cavernous nerve on the unilateral side. A pressure sensor was connected to record the ICP. After completion of ICP recording, the abdominal aorta was carefully exposed, and mean arterial pressure (MAP) was measured in the same animal using the same recording system. The ICP/MAP ratio was subsequently calculated.

### 4.23 Hematoxylin‐Eosin Staining

After the tissue samples were dewaxed and rehydrated, the following sequential staining steps were carried out: The samples were stained with hematoxylin for 5 min, then differentiated in 1% hydrochloric acid‐ethanol for 1 min, followed by bluing in ammonia water for 1 min, and finally stained with eosin for 1 min. After staining, the samples were dehydrated, mounted, and then observed under a microscope.

### 4.24 Masson Staining

The sample was immersed in hematoxylin staining solution for 5 min, and then stained with Ponceau acid‐fuchsin for 6 min. After that, the sample was soaked in a 1% phosphomolybdic acid solution for 1 min, followed by staining with a 2.5% aniline blue solution for 2 min. Finally, the sample was rinsed and differentiated with 1% glacial acetic acid.

### 4.25 Immunofluorescence

Permeabilization was carried out using Triton, followed by blocking with goat serum. The samples were then incubated with the primary antibody at 4°C overnight, and subsequently with the secondary antibody and DAPI at room temperature. Finally, the samples were observed under a fluorescence microscope.

### 4.26 Clinical Specimen Collection

The blood samples of DMED patients and the control group were collected from Xiangya Second Hospital of Central South University. The local ethics committee (LYEC2025‐0188) approved the ethical approval for the use of human blood samples. All patients' written informed consent was obtained before the collection of samples. We evaluated male patients with diabetes mellitus by iief5 scale, and defined patients with IIEF‐5 score less than 22 as DMED, while the control group was age‐matched healthy men. The plasma of the above population was collected for nontarget metabonomic detection.

### 4.27 Untargeted Metabolomics Analysis

Plasma samples were thawed on ice and extracted with pre‐chilled methanol/acetonitrile/water (2:2:1). After vortexing, ultrasonic treatment, and centrifugation, the supernatants were vacuum‐dried and reconstituted in acetonitrile/water (1:1) for LC‐MS analysis.

Metabolomic profiling was performed using UHPLC coupled to either a Triple TOF 6600 (AB SCIEX) or an Orbitrap Exploris 480 (Thermo Scientific) mass spectrometer. Chromatographic separation was achieved on a HILIC column under a standardized gradient elution program. Samples were analyzed in both positive and negative electrospray ionization (ESI) modes. QC samples were included to monitor instrument stability.

Raw data were converted to mzXML format and processed using XCMS for peak alignment and quantification. Features with > 50% missing values or RSD > 50% were filtered out. Missing values were imputed using the KNN algorithm, and identified metabolites were subjected to downstream statistical analysis.

### 4.28 Statistical Analysis

As previously described [52], statistical analyses were performed using GraphPad Prism 9. Comparisons between two groups were conducted using a two‐tailed unpaired Student's *t*‐test, while comparisons among multiple groups were analyzed using one‐way ANOVA followed by Tukey's post hoc test. Data are presented as mean ± SD, and the sample size (n) for each experiment is indicated in the Figure legends. A *p*‐value < 0.05 was considered statistically significant.

## Author Contributions

W.Y.G., M.X., and M.J.H. contributed to project administration, conceptualization, and source. D.Z.P. contributed to methodology and formal analysis. R.J.Z. contributed to methodology and formal analysis. R.L.L. contributed to methodology and formal analysis. Y.Q.Z., Z.H.O., Y.L.H., Z.X.D., Z.X., and H.S. contributed to formal analysis. J.X.W. and W.J.M. contributed to methodology. X.J. contributed to methodology, data curation, and resources.

## Ethics Statement

The local ethics committee (LYEC2025‐0188) approved the ethical approval for the use of human blood samples. All animal experiments were approved by the Animal Use and Care Committee of the Second Xiangya Hospital, Central South University (Approval No. 20250778), and were conducted in accordance with the institutional guidelines for the care and use of laboratory animals.

## Conflicts of Interest

The authors declare no conflicts of interest.

## Supporting information




**Supporting File**: advs75535‐sup‐0001‐SuppMat.docx.

## Data Availability

The data that support the findings of this study are available from the corresponding author upon reasonable request.
