# Supplementary material for: ZDHHC9‐Mediated Palmitoylation of ACSL4 Drives Ferroptosis in Diabetes Mellitus–Induced Erectile Dysfunction
Source: Adv Sci (Weinh). 2026 May 5;13(42):e17067. doi: 10.1002/advs.202517067 (PMC13335638; doi:10.1002/advs.202517067)
Supplement: Supplementary file 1 — Supporting File: advs75535‐sup‐0001‐SuppMat.docx. [file ADVS-13-e17067-s001.docx]

**ZDHHC9-Mediated Palmitoylation of ACSL4 Drives Ferroptosis in Diabetes Mellitus–Induced Erectile Dysfunction**

Wanyang Guo#^1,2,3,4^, Ming Xiao#^1,2,3,4^, Mengjun Huang#^1,2,3,4^, Dongzi Peng^1,2,3,4,5^,Ruijiang Zeng^1,2,3,4^, Ruilin Liu^1,2,3,4^, Yuanqiao Zhao^1,2,3,4^, Zhihan Ouyang^1,2,3,4^, Yulong Hong^1,2,3,4^, Zexian Ding^1,2,3,4^, Zhuo Xing^1,2,3,4^, Hao Su^1,2,3,4^, Jinxiang Wang^6,^*, Wenjun Mao^7,^*, Xin Jin^1,2,3,4,^*

^1^Department of Urology, The Second Xiangya Hospital, Central South University, Changsha, Hunan, 410011, China

^2^Key Laboratory of Diabetes Immunology (Central South University), Ministry of Education, National Clinical Research Center for Metabolic Disease, Changsha, China.

^3^FuRong Laboratory, Changsha, Hunan,410000, China.

^4^Biobank of the Second Xiangya Hospital of Central South University

^5^Department of Gastroenterology, The Second Xiangya Hospital, Central South University, Changsha, Hunan,410011, China.

^6^Department of Urology, Kidney and Urology Center, Pelvic Floor Disorders Center, The Seventh Affiliated Hospital, Sun Yat-sen University, Shenzhen, Guangdong, 518107, China

^7^Department of Thoracic Surgery, The Affiliated Wuxi People’s Hospital of Nanjing Medical University, Wuxi People’s Hospital, Wuxi Medical Center, Nanjing Medical University, Wuxi, Jiangsu, China.

***Corresponding authors**: Xin Jin (jinxinxy2@csu.edu.cn); Wenjun Mao(maowenjun1@njmu.edu.cn); Jinxiang Wang(wangjx258@mail.sysu.edu.cn)

**Supplementary Figures**

**
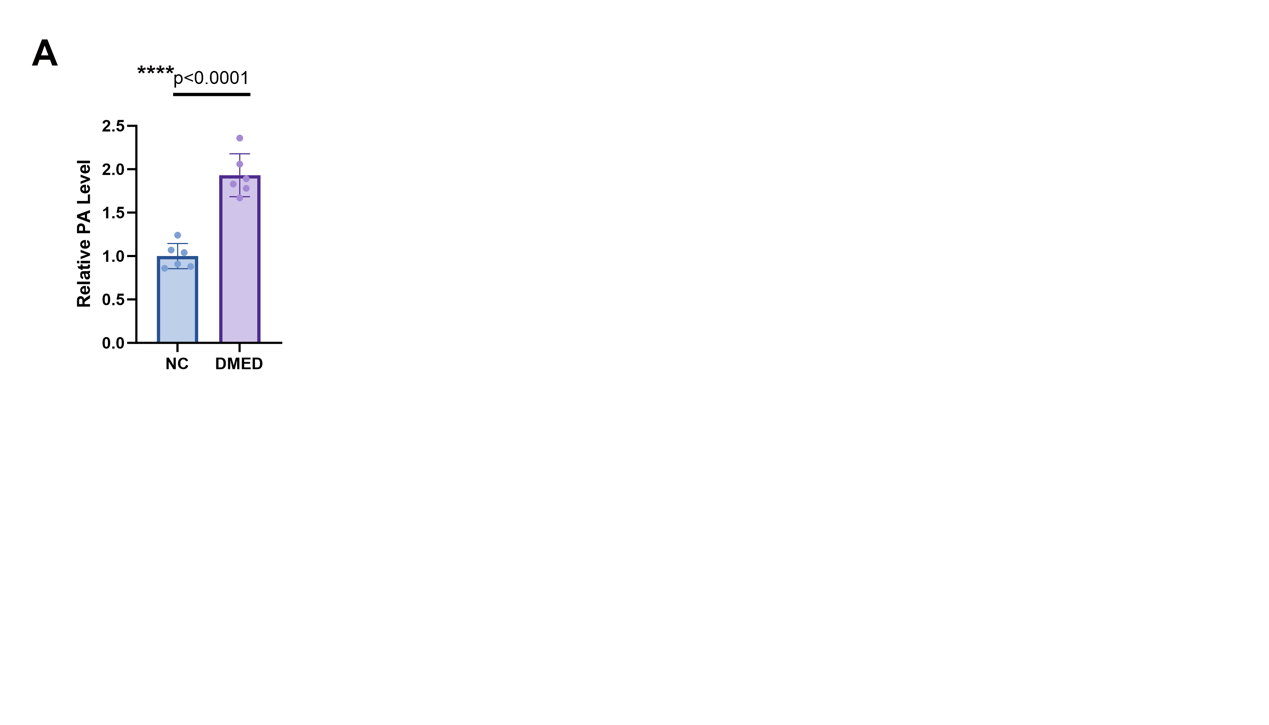
**

**Figure S1. Increased PA levels in the corpus cavernosum of mice**

A, PA levels in the penile cavernous tissue of mice. n=6. Bar charts are presented as mean ± SD. Unpaired two-sided Student’s t-test was performed. p-values have been indicated in the figures, and p < 0.05 is considered statistically significant.

**
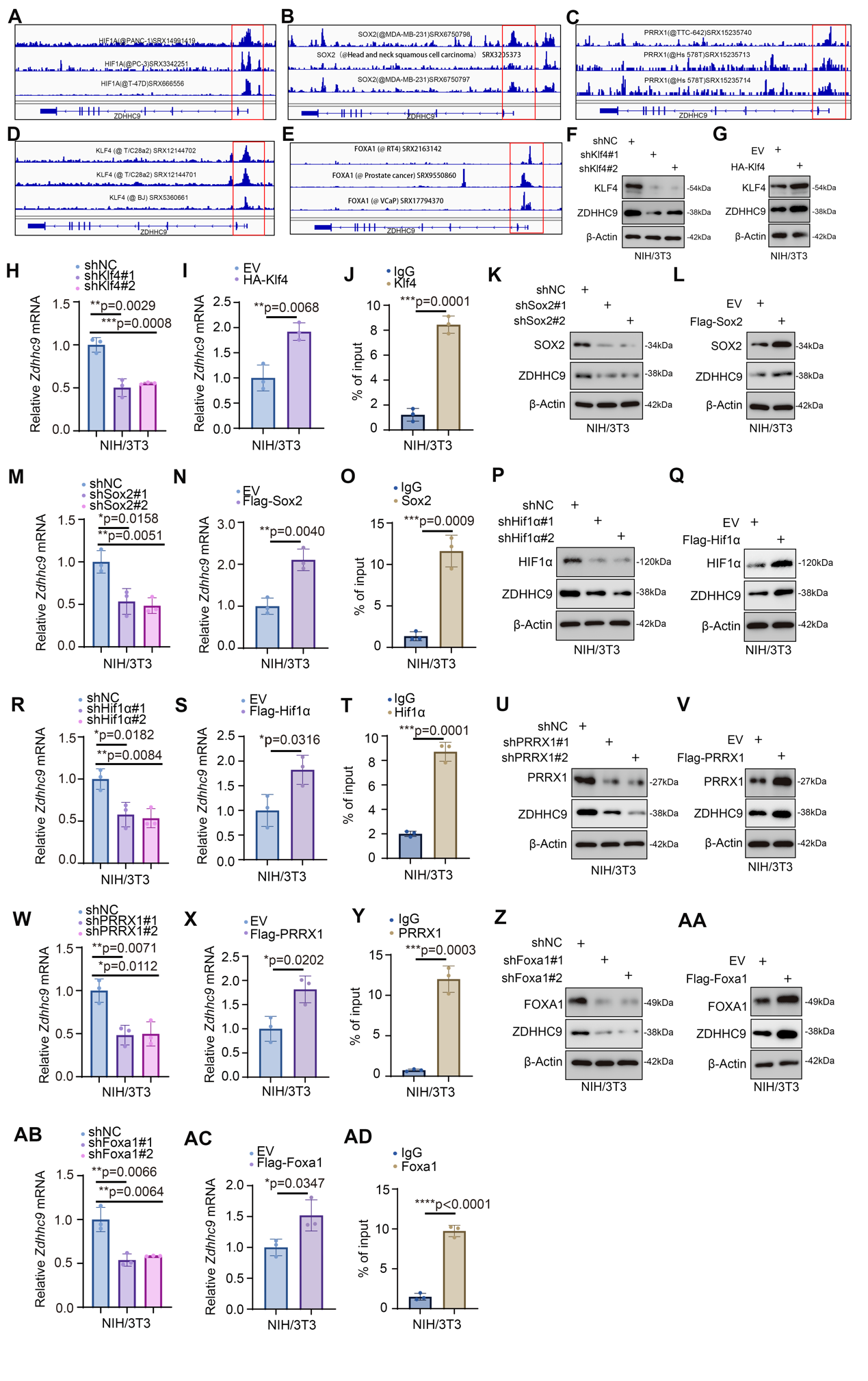
**

**Figure S2. ChIP-qPCR and Western blotting confirmed that five transcription factors downstream of PI3K-AKT regulate ZDHHC9 transcription**

A-E, Transcription factors binding to the *ZDHHC9* promoter identified from the ChIP-Atlas database (https://chip-atlas.org/). F, Western blot analysis of effects of shRNA-mediated knockdown of *Klf4* on ZDHHC9 in NIH/3T3 cells(n=3). G, Western blot analysis of effects of *Klf4* overexpression on ZDHHC9 in NIH/3T3 cells(n=3). H, RT-qPCR analysis of effects of shRNA-mediated knockdown of *Klf4* on *Zdhhc9* in NIH/3T3 cells(n=3). I, RT-qPCR analysis of effects of *Klf4* overexpression on *Zdhhc9* in NIH/3T3 cells(n=3). J, The ChIP-qPCR assay was used to confirm the interaction between *Klf4* and *Zdhhc9* in NIH/3T3 cells(n=3). K, Western blot analysis of effects of shRNA-mediated knockdown of *Sox2* on ZDHHC9 in NIH/3T3 cells(n=3). L, Western blot analysis of effects of *Sox2* overexpression on ZDHHC9 in NIH/3T3 cells(n=3). M, RT-qPCR analysis of effects of shRNA-mediated knockdown of *Sox2* on *Zdhhc9* in NIH/3T3 cells(n=3). N, RT-qPCR analysis of effects of effects of *Sox2* overexpression on *Zdhhc9* in NIH/3T3 cells(n=3). O, The ChIP-qPCR assay was used to confirm the interaction between *Sox2* and *Zdhhc9* in NIH/3T3 cells(n=3). P, Western blot analysis of effects of shRNA-mediated knockdown of *Hif1α* on ZDHHC9 in NIH/3T3 cells(n=3). Q, Western blot analysis of effects of *Hif1α* overexpression on ZDHHC9 in NIH/3T3 cells(n=3). R, RT-qPCR analysis of effects of shRNA-mediated knockdown of *Hif1α* on *Zdhhc9* in NIH/3T3 cells(n=3). S, RT-qPCR analysis of effects of effects of *Hif1α* overexpression on *Zdhhc9* in NIH/3T3 cells(n=3). T, The ChIP-qPCR assay was used to confirm the interaction between *Hif1α* and *Zdhhc9* in NIH/3T3 cells(n=3). U, Western blot analysis of effects of shRNA-mediated knockdown of *Prrx1* on ZDHHC9 in NIH/3T3 cells(n=3). V, Western blot analysis of effects of *Prrx1* overexpression on ZDHHC9in NIH/3T3 cells(n=3). W, RT-qPCR analysis of effects of shRNA-mediated knockdown of *Prrx1* on *Zdhhc9* in NIH/3T3 cells(n=3). X, RT-qPCR analysis of effects of effects of *Prrx1* overexpression on *Zdhhc9* in NIH/3T3 cells(n=3). Y, The ChIP-qPCR assay was used to confirm the interaction between *Prrx1* and *Zdhhc9* in NIH/3T3 cells(n=3). Z, Western blot analysis of effects of shRNA-mediated knockdown of *Foxa1* on ZDHHC9 in NIH/3T3 cells(n=3). AA, Western blot analysis of effects of *Foxa1* overexpression on ZDHHC9in NIH/3T3 cells(n=3). AB, RT-qPCR analysis of effects of shRNA-mediated knockdown of *Foxa1* on *Zdhhc9* in NIH/3T3 cells(n=3). AC, RT-qPCR analysis of effects of effects of *Foxa1* overexpression on *Zdhhc9* in NIH/3T3 cells(n=3). AD, The ChIP-qPCR assay was used to confirm the interaction between *Foxa1* and *Zdhhc9* in NIH/3T3 cells(n=3). Bar charts(H,I,J,M,N,O,R,S,T,W,X,Y,AB,AC,AD) are presented as mean ± SD. All experiments were performed with at least three biologically independent cell/mouse samples with similar results. Unpaired two-sided Student’s *t*-test (I,J,N,O,S,T,X,Y,AC,AD) and one-way ANOVA with Tukey’s post hoc test (H,M,R,W,AB) were performed. p-values have been indicated in the figures, and p < 0.05 is considered statistically significant.

**
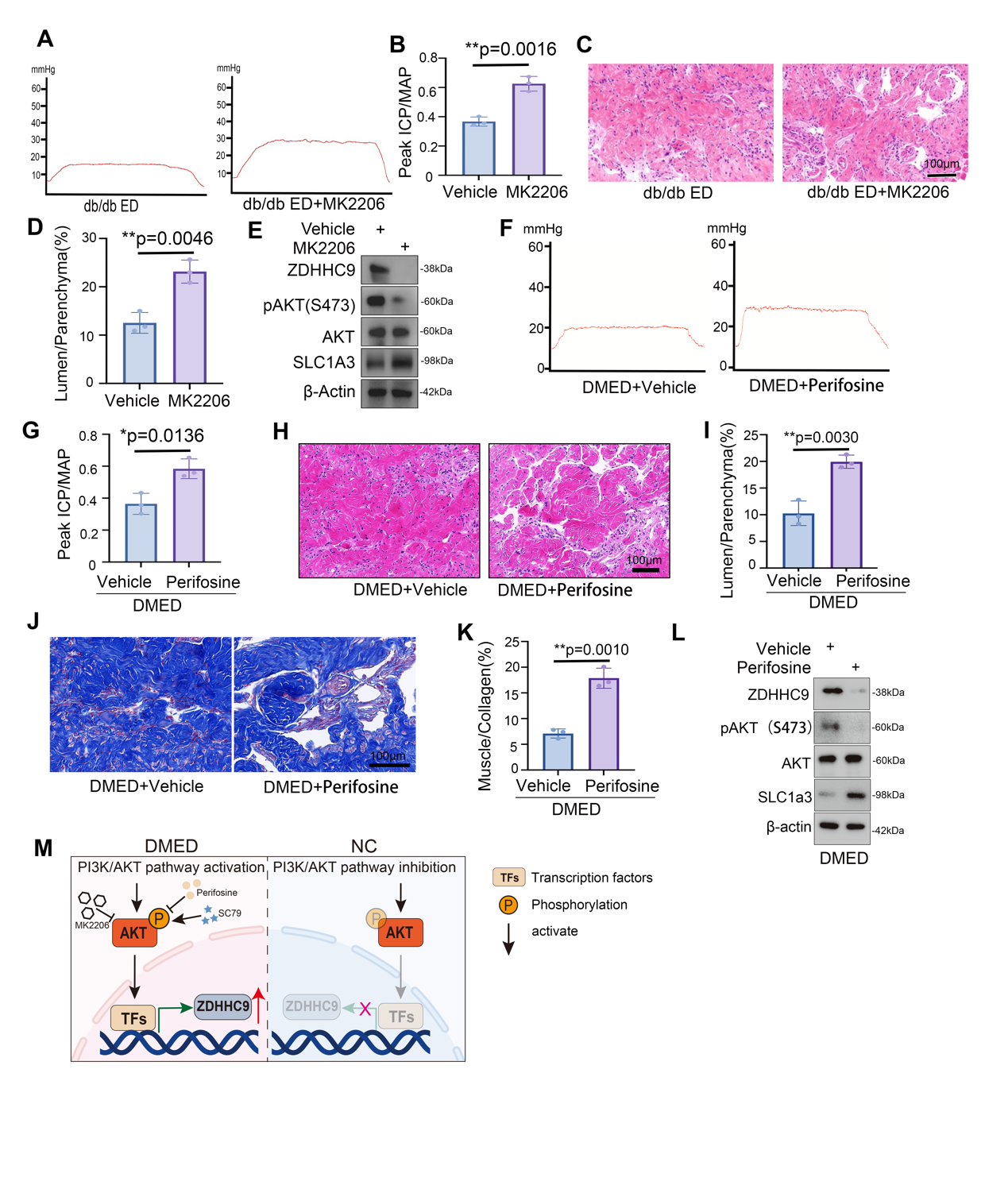
**

**Figure S3. PI3K/AKT pathway hyperactivation and ZDHHC9-driven pathogenesis in DMED.**

A-B, Representative images of ICP and quantification of ICP/MAP ratio (n=3). C-D, Morphology and quantification of the corpus cavernosum was assessed by H&E. Scale bars, 100 µm(n=3). E, Western blot analysis of MK2206 on Zdhhc9 and PI3K/AKT signaling in vivo(n=3). F-G, Representative images of ICP and quantification of ICP/MAP ratio(n=3). H-I, Morphology and quantification of the corpus cavernosum was assessed by H&E. Scale bars, 100 µm(n=3). J-K, Collagen (blue) and smooth muscle (red) were evaluated by Masson staining. Scale bars, 100 µm(n=3). L, Western blot analysis of effects of Perifosine treatment on Zdhhc9 and PI3K-AKT pathway in vivo(n=3). M, Schematic illustration of the PI3K-AKT signaling pathway regulating ZDHHC9 expression. Bar charts(B,D,J,I,K) are presented as mean ± SD. All experiments were performed with at least three biologically independent cell/mouse samples with similar results. Unpaired two-sided Student’s *t*-test (B,D,J,I,K) were performed. p-values have been indicated in the figures, and p < 0.05 is considered statistically significant.

**
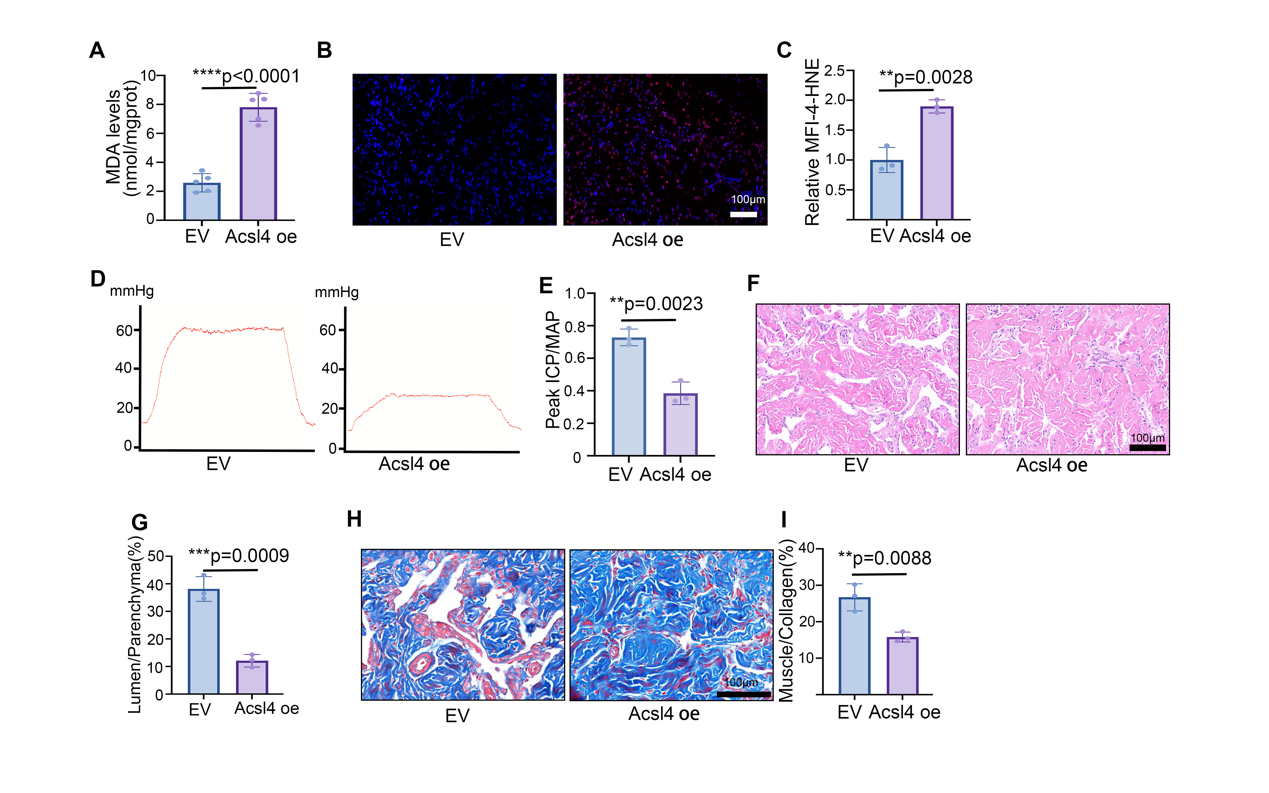
**

**Figure S4.** **Acsl4 overexpression in murine corpus cavernosum exacerbates DMED.**

A, Measurement of MDA Levels in Corpus Cavernosum(n=5). B-C, Immunofluorescence staining (B) and MFI quantification of 4-NHE level(C) (n=3). D-E, Representative images of ICP and quantification of ICP/MAP ratio (n=3). F-G, Morphology and quantification of the corpus cavernosum was assessed by H&E. Scale bars, 100 µm (n=3). H-I, Collagen (blue) and smooth muscle (red) were evaluated by Masson staining. Scale bars, 100 µm (n=3). Bar charts(A,C,E,G,I) are presented as mean ± SD. All experiments were performed with at least three biologically independent cell/mouse samples with similar results. Unpaired two-sided Student’s *t*-test (A,C,E,G,I) were performed. p-values have been indicated in the figures, and p < 0.05 is considered statistically significant.

**
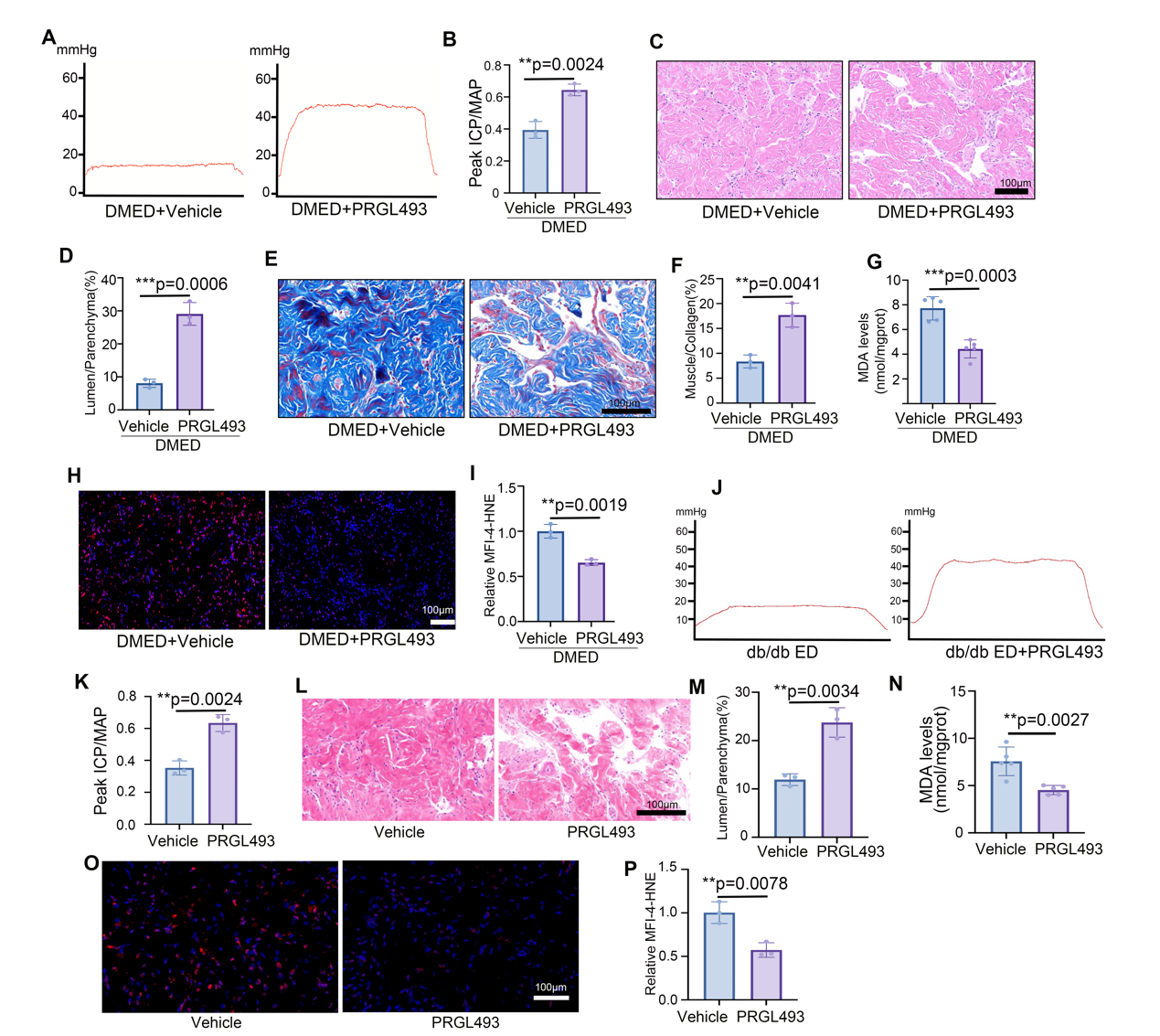
**

**Figure S5. Inhibition of ACSL4 enzyme activity alleviates DMED.**

A-B, Representative images of ICP and quantification of ICP/MAP ratio (n=3). C-D, Morphology and quantification of the corpus cavernosum was assessed by H&E. Scale bars, 100 µm (n=3). E-F, Collagen (blue) and smooth muscle (red) were evaluated by Masson staining. Scale bars, 100 µm (n=3). G, Measurement of MDA Levels in Corpus Cavernosum (n=5). H-I, Immunofluorescence staining (H) and MFI quantification of 4-NHE level(I) (n=3). J-K, Representative images of ICP and quantification of ICP/MAP ratio (n=3). L-M, Morphology and quantification of the corpus cavernosum was assessed by H&E. Scale bars, 100 µm. (n=3). N, Measurement of MDA Levels in Corpus Cavernosum (n=5). O-P, Immunofluorescence staining (O) and MFI quantification of 4-NHE level(P) (n=3). Bar charts(B,D,F,G,I,K,M,N,P) are presented as mean ± SD. All experiments were performed with at least three biologically independent cell/mouse samples with similar results. Unpaired two-sided Student’s *t*-test (B,D,F,G,I,K,M,N,P) were performed. p-values have been indicated in the figures, and p < 0.05 is considered statistically significant.

**
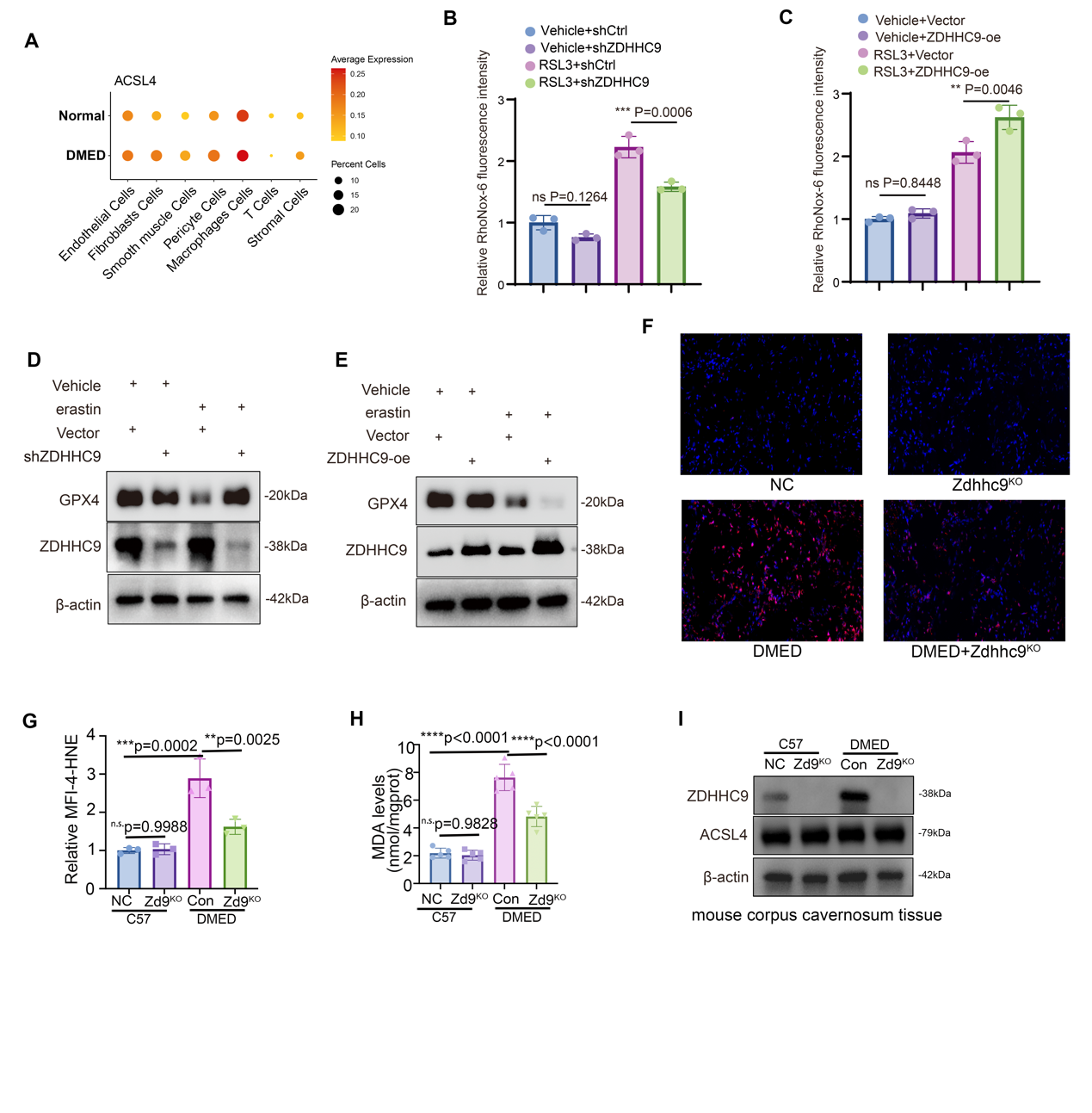
**

**Figure S6. *Zdhhc9*^ko^ suppressed ferroptosis but didn’t change ACSL4 protein level in mouse corpus cavernosum tissue.**

A, Expression of *ACSL4* in each group based on single-cell data analysis of the human corpus cavernosum (http://malehealthatlas.cn/). B-C, NIH/3T3 cells with ZDHHC9 knockdown (B) or overexpression (C) were treated as indicated, followed by RhoNox-6 fluorescence analysis of intracellular ferrous iron levels. D-E, Western blot analysis of GPX4 expression in NIH/3T3 cells with ZDHHC9 knockdown (D) or overexpression (E) following the indicated treatments. F-G, Immunofluorescence staining (F) and MFI quantification of 4-NHE level(G) (n=3). H, Measurement of MDA Levels in Corpus Cavernosum(n=5). I, Western blot analysis of ZDHHC9 and ACSL4 in corpus cavernosum tissue of normal or DMED Zdhhc9^ko^ mice(n=3). Bar charts(B,C,G,H) are presented as mean ± SD. All experiments were performed with at least three biologically independent cell/mouse samples with similar results. One-way ANOVA with Tukey’s post hoc test (B,C,G,H) were performed. p-values have been indicated in the figures, and p < 0.05 is considered statistically significant.

**
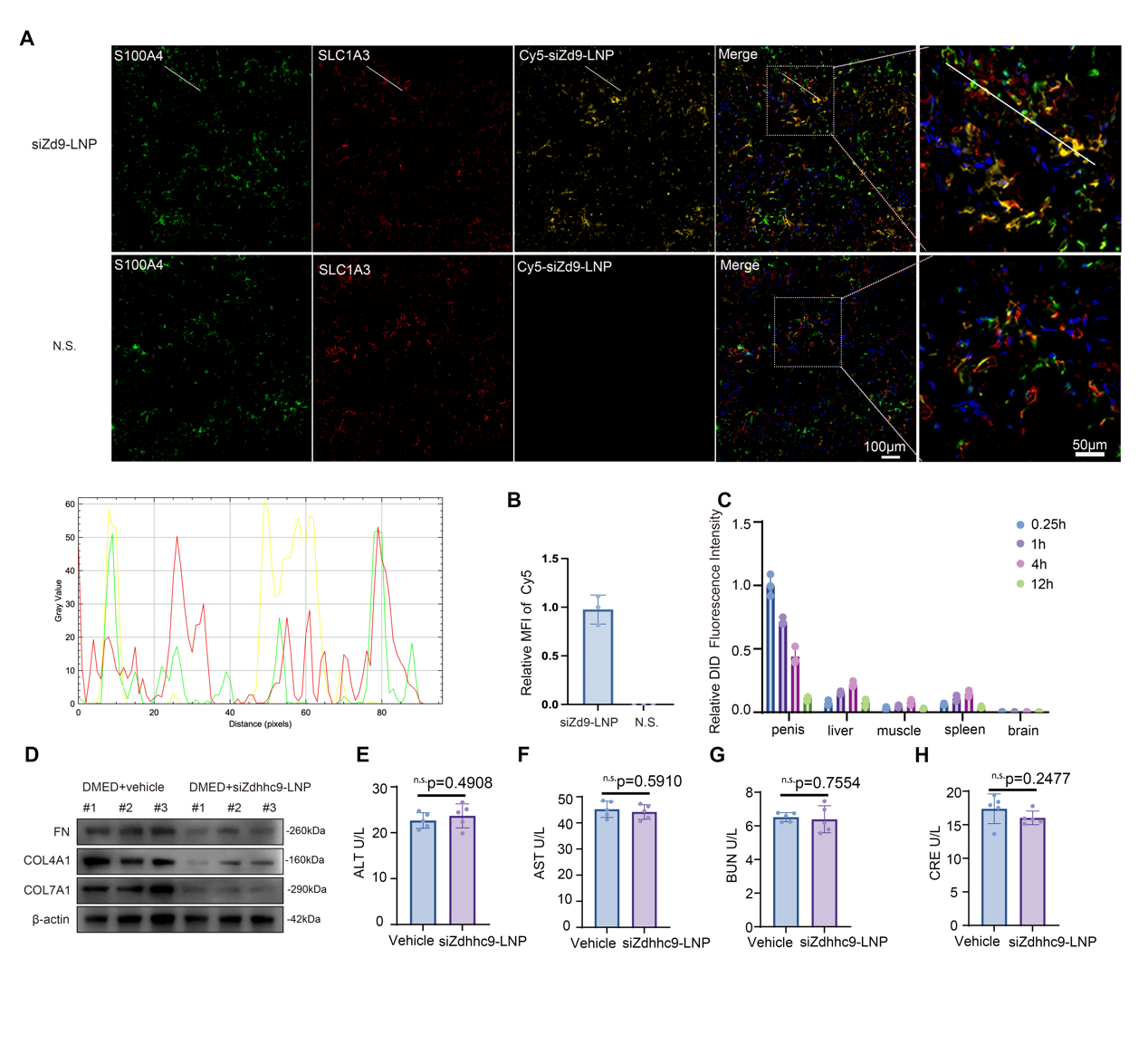
**

**Figure S7. LNP treatment did not induce hepatotoxicity or nephrotoxicity and are exclusively mediated through ZDHHC9 inhibition.**

A-B, Corpus cavernosum distribution and quantification of of Cy5-siZdhhc9-LNP following the indicated administration. Scale bar, 100 µm (n=3). C, Time-course biodistribution of DiD-labeled siZdhhc9-LNPs. Tissues were harvested at 0.25, 1, 4, and 12 h after intracavernosal injection, weighed, homogenized, and fluorescence intensity in the tissue homogenates was measured using a multimode microplate reader. (n=3). D, Western blot analysis of FN, COL4A1 and COL7A1 in corpus cavernosum tissue of DMED mice with/without siZdhhc9-LNP administration (n=3). E, Serum ALT levels (n=5). F, Serum AST levels (n=5). G, Serum BUN levels (n=5). H, Serum CRE levels (n=5). Bar charts(B,E,F,G,H) are presented as mean ± SD. All experiments were performed with at least three biologically independent cell/mouse samples with similar results. Unpaired two-sided Student’s *t*-test (B,E,F,G,H) were performed. p-values have been indicated in the figures, and p < 0.05 is considered statistically significant.


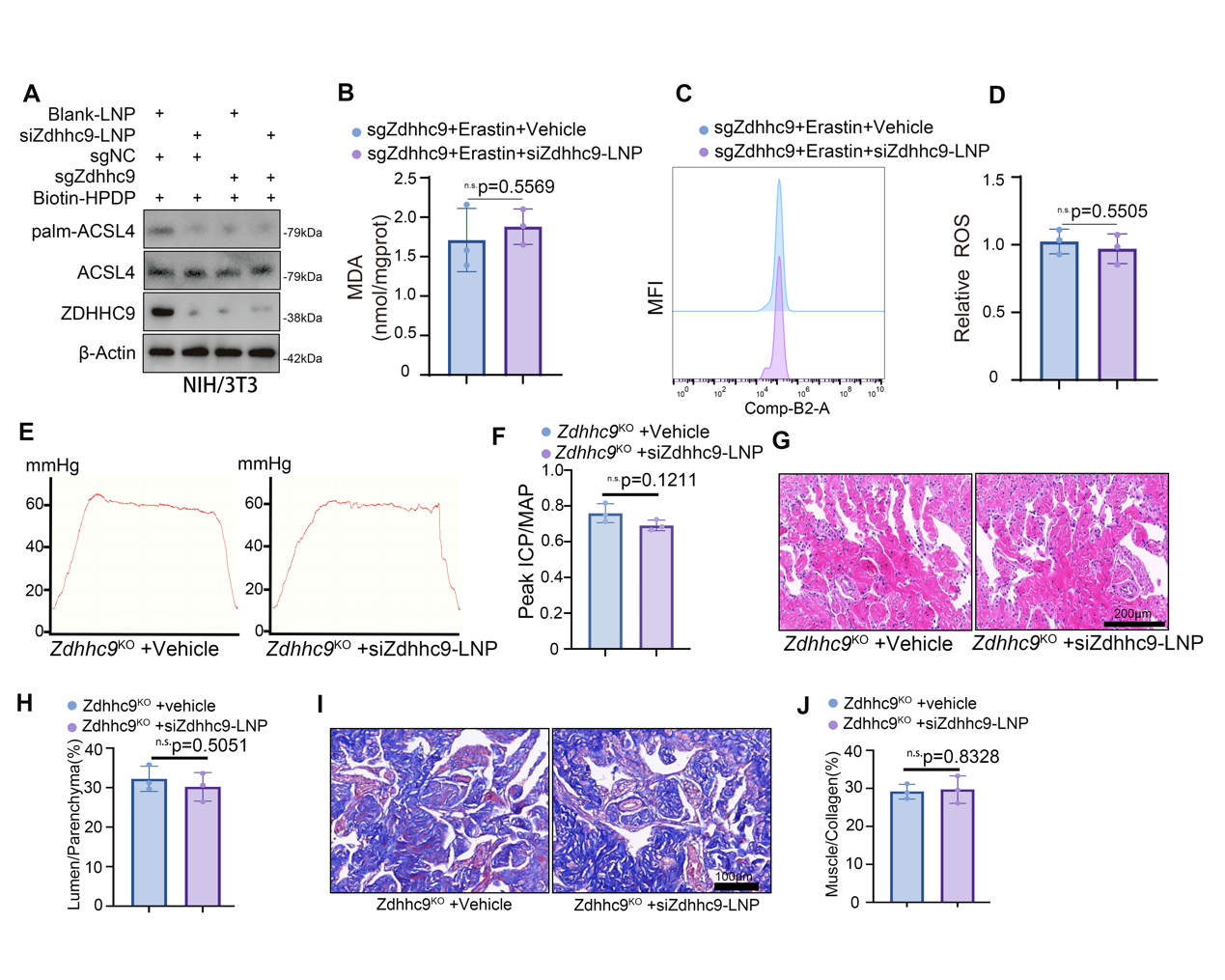


**Figure S8. *Zdhhc9*^ko^ mice exhibited no therapeutic response to LNP treatment in ZDHHC9-deficient cells and mice.**

A, Western blot analysis of ZDHHC9 expression and ABE assay of ACSL4 palmitoylation in NIH/3T3 cells following indicated treatments(n=3). B–D, NIH/3T3 cells were treated as indicated, followed by analysis of MDA (B) (n=3) and ROS (C, D) (n=3). E-F, Representative images of ICP and quantification of ICP/MAP ratio(n=3). G-H, Morphology of the corpus cavernosum assessed by H&E staining (scale bars, 200 µm) (n=3). I-J, Collagen (blue) and smooth muscle (red) were evaluated by Masson staining. Scale bars, 100 µm(n=3). Bar charts(B,D,F,H,J) are presented as mean ± SD. All experiments were performed with at least three biologically independent cell/mouse samples with similar results. Unpaired two-sided Student’s *t*-test (B,D,F,H,J) were performed. p-values have been indicated in the figures, and p < 0.05 is considered statistically significant.

**Supplementary Tables**

**Table S1. The siRNA and sgRNA sequences.**

| siZdhhc9 | 5’- AGAUUGUGAAACUGAAAUACU-3‘’ |
| --- | --- |
| shKlf4#1 | 5’- GGTCATCAGTGTTAGCAAA-3‘’ |
| shKlf4#2 | 5’- GGAAGAGTTCAAGAGACAA-3‘’ |
| shSox2#1 | 5’-CGTTCTAGATTGTACTAAA-3 |
| shSox2#2 | 5’-GGTTTGTAATATTTCTGTA-3 |
| shHif1α#1 | 5’-GGAGGACGATGAACATCAA-3 |
| shHif1α#2 | 5’-GGATGATGATTTCCAGTTA-3 |
| shPrrx1#1 | 5’- CATTGTTTGTGCTAGAATA-3 |
| shPrrx1#2 | 5’- GGCACAAAGTATAGAGATA-3 |
| shFoxa1#1 | 5’- CGATGTGATTTGTAATTAA-3 |
| shFoxa1#2 | 5’- GGTTAAATTGACAAATTAA-3 |
| sgZdhhc1 #1 | 5’- GAAAAGAAGCCTGCACTCTGAT-3’ |
| sgZdhhc1 #2 | 5’-CACAGTGTGGCATCTGCTTTAT -3’ |
| sgZdhhc2 #1 | 5’-TATGCAGAGAAAGAATTGCTGG -3’ |
| sgZdhhc2 #2 | 5’-TCTCCTGCTCTTGGAAAATCTC -3’ |
| sgZdhhc3 #1 | 5’-ACTGTATGATGGTTACCCCTGC -3’ |
| sgZdhhc3 #2 | 5’-TAGGAGATTTTCCCAACAATGG -3’ |
| sgZdhhc4 #1 | 5’-CTTGTGCTTTGCTCTGTACCTG -3’ |
| sgZdhhc4 #2 | 5’-ATAACGTCCATCCCTTCTCTCA -3’ |
| sgZdhhc5 #1 | 5’- CCAAACTGACTCTTGTCCCAGT -3’ |
| sgZdhhc5 #2 | 5’- ATTCCCAAACTGACTCTTGTCC -3’ |
| sgZdhhc6 #1 | 5’- CAAGAAAAATCCTTCCCTGATG -3’ |
| sgZdhhc6 #2 | 5’-CTTGTGGTATTGGCCCTTACAT -3’ |
| sgZdhhc7 #1 | 5’-AACCTCACTGTTCTCACCACCT -3’ |
| sgZdhhc7 #2 | 5’-GTGGGAAGGAATGAAGTCTGTC -3’ |
| sgZdhhc8 #1 | 5’-GTGGGATTTCCTCCACTGC -3’ |
| sgZdhhc8 #2 | 5’-TTCATGTTCTTGCCTCAACTGT -3’ |
| sgZdhhc9 #1 | 5’- TGGAGACTTTGGACTCAAACAA -3’ |
| sgZdhhc9 #2 | 5’- ATTAGCCCACACTGCCTGTATT -3’ |
| sgZdhhc11 #1 | 5’- CAGCATACTCTCATGCAGGTTC -3’ |
| sgZdhhc11 #2 | 5’- GTACACATGGCTATTGCTCCAA -3’ |
| sgZdhhc12 #1 | 5’- CACGACTACCTGGGAGTTCATA -3’ |
| sgZdhhc12 #2 | 5’- ACTAAGCCTAAACTGCGCTCTG -3’ |
| sgZdhhc13 #1 | 5’- GAGGGAGGGAAAGGATAGAAGA -3’ |
| sgZdhhc13 #2 | 5’- AGTGCAGTTGATAAGCTTTTGG -3’ |
| sgZdhhc14 #1 | 5’- GCCAGAATCTCCATTAAGCAAC -3’ |
| sgZdhhc14 #2 | 5’- AGTACAGTCAGATCAGCACCCA -3’ |
| sgZdhhc15 #1 | 5’-TTCCTGTAGCTGTGACTCAGGC -3’ |
| sgZdhhc15 #2 | 5’-TTCCTGTAGCTGTGACTCAGGC -3’ |
| sgZdhhc16 #1 | 5’-AGTTTGACTCCTGTCTTCCAGC -3’ |
| sgZdhhc16 #2 | 5’-AGTTTGACTCCTGTCTTCCAGC -3’ |
| sgZdhhc17 #1 | 5’-GTTTTTAAGTGTGGCAGCCATT -3’ |
| sgZdhhc17 #2 | 5’-TGAGGTGACTGGAAGGGTAAAT -3’ |
| sgZdhhc18 #1 | 5’-GGATGTGTGTCTCTGCATTAGC -3’ |
| sgZdhhc18 #2 | 5’-TAACACAGGCAGCTCCACATAC-3’ |
| sgZdhhc19 #1 | 5’-CCACGTCGTCCTAAACACTGTA -3’ |
| sgZdhhc19 #2 | 5’-AGCTTGCTCAGTAAGATCAGGG -3’ |
| sgZdhhc20 #1 | 5’-GGCAGTCAGCAGAGGAACAT -3’ |
| sgZdhhc20 #2 | 5’-CTTGTGGTCCTACAGAATCGTG -3’ |
| sgZdhhc21 #1 | 5’-GATCCTGTGGTTCATTCCTTTC -3’ |
| sgZdhhc21 #2 | 5’-GGATGTGTGTGCTCTCTCTCTG -3’ |
| sgZdhhc22 #1 | 5’-CTGACATGTTCGTCATCCTCAT -3’ |
| sgZdhhc22 #2 | 5’-CGCAAGAACTTACAGGAGGTCT -3’ |
| sgZdhhc23 #1 | 5’-TTTACGGAATATCGCTGACCTT -3’ |
| sgZdhhc23 #2 | 5’-TCACAGAAGGAAAGAACAGACG -3’ |
| sgZdhhc24 #1 | 5’-AAAGTGTCTCTGGCCCAGTTT -3’ |
| sgZdhhc24 #2 | 5’-CTGTGTCTCCTGCTTCACTCTG -3’ |

**Table S2. The primer sequences for RT-qPCR.**

| Gene | Forward (5′ - 3′) | Reverse (3′ - 5′) |
| --- | --- | --- |
| Actb | CGCAGCCACTGTCGAGTC | GTCATCCATGGCGAACTGGT |
| Zdhhc9 | CCTGTCAGGGAGAAGTCGCT | CAAACATTATCAAGAGCCCTGAACA |
| ACTB | GATCATTGCTCCTCCTGAGC | GGGCCGGACTCGTCATA |
| ZDHHC9 | GTTGGGTCTCCGTGGTTCAG | CCAATTGCTAGCCCTGGAAGA |

**Table S3. The primer sequences for ChIP-qPCR.**

| Gene (mouse) | Forward (5′ - 3′) | Reverse (3′ - 5′) |
| --- | --- | --- |
| Zdhhc9 | AGCATGCGATTCCTAAGGGC | TCTCATGGCCCTATCAGCTCT |
